# Crosstalk between m6A modification and alternative splicing during cancer progression

**DOI:** 10.1002/ctm2.1460

**Published:** 2023-10-18

**Authors:** Zhi‐Man Zhu, Fu‐Chun Huo, Jian Zhang, Hong‐Jian Shan, Dong‐Sheng Pei

**Affiliations:** ^1^ Department of Pathology Xuzhou Medical University Xuzhou Jiangsu China; ^2^ Department of Respiratory Medicine Second Affiliated Hospital of Xuzhou Medical University Xuzhou Jiangsu China; ^3^ Department of Orthopedics The Affiliated Jiangning Hospital with Nanjing Medical University Nanjing Jiangsu China

**Keywords:** alternative splicing, cancer progression, m6A modification, RNA metabolism

## Abstract

**Background:**

N6‐methyladenosine (m6A), the most prevalent internal mRNA modification in eukaryotes, is added by m6A methyltransferases, removed by m6A demethylases and recognised by m6A‐binding proteins. This modification significantly influences carious facets of RNA metabolism and plays a pivotal role in cellular and physiological processes.

**Main body:**

Pre‐mRNA alternative splicing, a process that generates multiple splice isoforms from multi‐exon genes, contributes significantly to the protein diversity in mammals. Moreover, the presence of crosstalk between m6A modification and alternative splicing, with m6A modifications on pre‐mRNAs exerting regulatory control, has been established. The m6A modification modulates alternative splicing patterns by recruiting specific RNA‐binding proteins (RBPs) that regulate alternative splicing or by directly influencing the interaction between RBPs and their target RNAs. Conversely, alternative splicing can impact the deposition or recognition of m6A modification on mRNAs. The integration of m6A modifications has expanded the scope of therapeutic strategies for cancer treatment, while alternative splicing offers novel insights into the mechanistic role of m6A methylation in cancer initiation and progression.

**Conclusion:**

This review aims to highlight the biological functions of alternative splicing of m6A modification machinery and its implications in tumourigenesis. Furthermore, we discuss the clinical relevance of understanding m6A‐dependent alternative splicing in tumour therapies.

## Graphical abstract

1. m6A modification regulating alternative splicing reveals a strong link between RNA epitranscriptomic modification and genomic complexity.

2. Alternative splicing of m6A modification machinery endows tumor cells with specific splicing patterns and characteristics.

3. The combination of alternative splicing with m6A modification provides novel prognostic models and therapeutic targets for individual cancer.

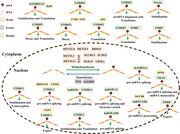

## 1 INTRODUCTION

Eukaryotic RNAs boast an impressive array of over 170 chemical modifications, encompassing N1‐methyladenosine, N6‐methyladenosine (m6A), 5‐methylcytosine, N7‐methylguanosine, hydroxymethylcytosine and pseudo‐uridine.^1^ Among these, m6A modification stands out as the most abundantly and evolutionarily conserved transcription alteration. Extensive research has comprehensively analysed its influence on various aspects of mRNA metabolism, including transcription, translation, decay, stability, splicing and localisation.^2^ The development of m6A‐immunoprecipitation and sequencing (MeRIP‐seq) techniques has facilitated a comprehensive exploration of the pivotal roles played by the m6A regulatory network in post‐transcriptional regulation, cellular processes and biological functions.^3^, ^4^, ^5^ Notably, alterations in m6A modification have been associated with RNA metabolic disorders and affect various human diseases, including cancer, obesity, diabetes, neurological disorders and infertility.^3^, ^4^, ^5^ Currently, the dysregulation of m6A modification has surfaced in the majority of human tumour types. Primarily, it exerts its impact on the post‐transcriptional modification and expression regulation of target transcripts by modulating the m6A levels of oncogenes or tumour suppressor genes.^6^, ^7^, ^8^ Exploring the molecular mechanism underpinning m6A modification in tumour progression holds immense clinical significance and value, offering potential avenues for anti‐tumour therapy.^9^, ^10^

RNA splicing is an essential eukaryotic process governing gene expression. It involves the excision of non‐coding regions, introns and long non‐coding (lnc) RNAs from pre‐mRNA, followed by the joining of exons to form mature mRNA.^11^ Constitutive pre‐mRNA splicing mainly involves intron removal and exon connection to ensure gene expression stability. Conversely, alternative splicing yields multiple transcripts and protein isoforms from a single gene,^11^ occasionally generating non‐coding RNAs as byproducts.^11^ Alternative splicing augments gene expression complexity and protein function diversity,^12^ contributing significantly to normal physiology while also being implicated in various diseases, such as Parkinson's disease, Alzheimer's disease, spinal muscular atrophy, familial dysautonomia, cystic fibrosis, Frasier syndrome and cancer initiation and progression.^13^, ^14^ In tumour cells, alternative splicing plays a crucial role in the development of diverse malignant phenotypes.^15^ Tumour‐specific alternative splicing holds promise as potential diagnostic or prognostic markers and opens novel avenues for targeted therapeutic strategies against tumours.

Recently, increasing evidence has emerged that unveils the regulatory role of alternative splicing of m6A modification machinery. Notably, deficiency of METTL3 has been demonstrated to significantly impact the alternative splicing patterns of thousands of genes.^16^, ^17^, ^18^, ^19^ During alternative splicing events, m6A peaks exhibit enrichment in exons and introns, with m6A‐modified exons displaying trends toward retention in mRNAs following splicing.^17^, ^18^ These findings highlight the significant contributions of m6A methylation to alternative splicing. This review emphasises the underlying mechanisms and functions of alternative splicing of m6A machinery and its implications in tumourigenesis. Furthermore, we explore the therapeutic potential of targeting m6A‐dependent alternative splicing in tumour therapy.

## 2 REGULATION OF M6A MODIFICATION

**FIGURE 1:**
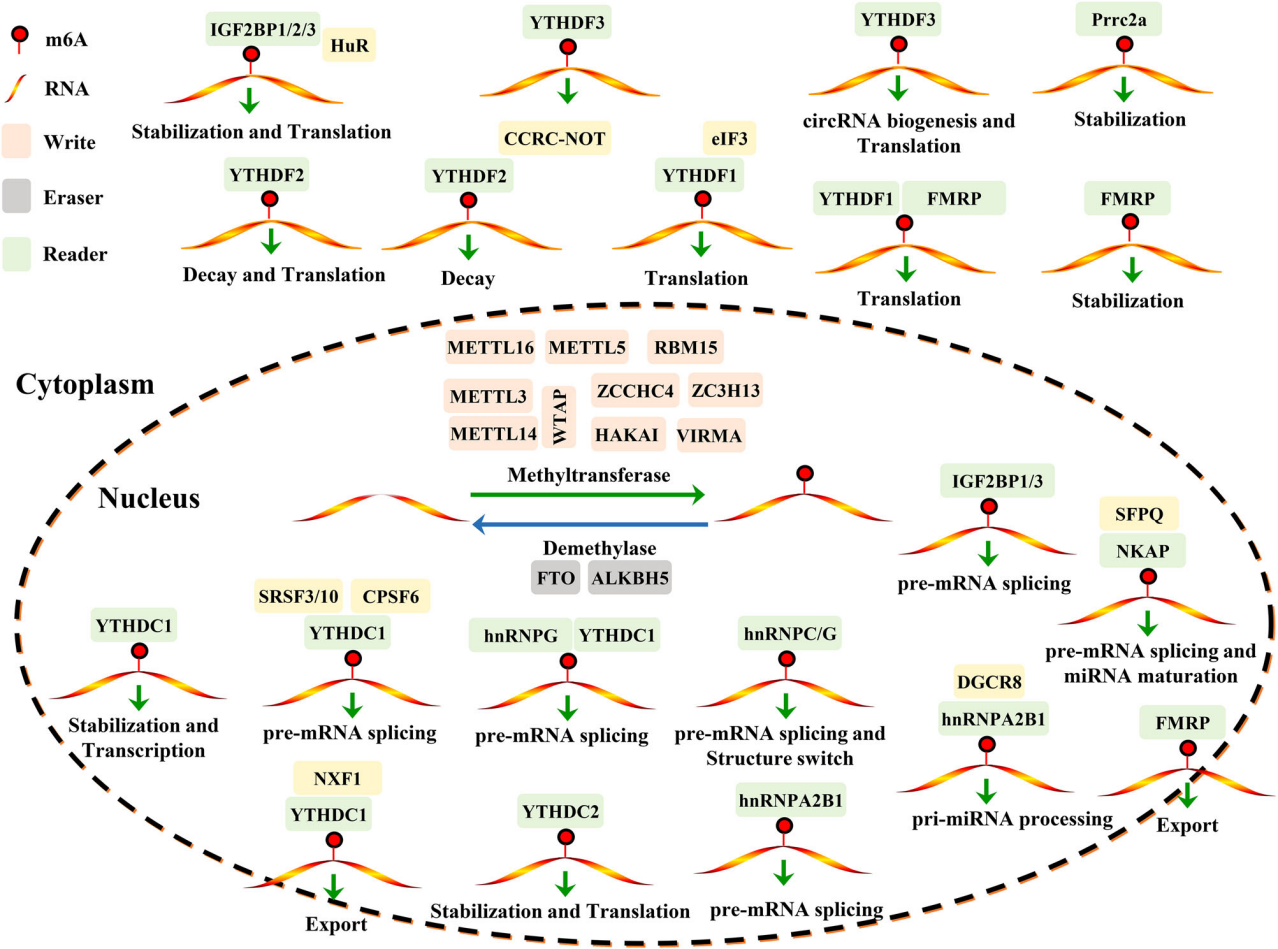
Regulation of the m6A modification. The m6A RNA modification is reversibly regulated by m6A methyltransferases (writers), m6A demethylases (erasers) and m6A‐binding proteins (readers). The writers are responsible for adding methyl groups to the N6 position of adenosine of specific RNAs, whereas erasers remove the methyl groups. The readers, on the other hand, are RBPs that can recognise and interpret the m6A modification, thereby affecting various diverse of RNA metabolism such as mRNA translation, decay, stability, splicing and localisation.

The m6A RNA modification is reversibly regulated by m6A methyltransferases (writers), m6A demethylases (erasers) and m6A‐binding proteins (readers).^20^(Figure 1). Writers are responsible for the addition of a methyl group to the N6 position of adenosine on target RNAs, whereas erasers remove the methyl group.^20^ Readers recognise and decipher the m6A modification, thereby instigating various functional consequences for the targeted RNAs.^20^ The deposition of m6A is catalysed by the methyltransferase complex (MTC), comprising essential methyltransferases such as METTL3, METTL14, METTL16, WTAP, VIRMA, ZC3H13, RBM15 and its paralog (RBM15B).^20^ The m6A MTC is responsible for introducing the m6A modification to the N6 position of adenosine. This process entails the transfer of methyl groups from S‐adenosylmethionine (SAM) through the methyltransferase enzyme. METTL3, functioning as the catalytic core of the m6A MTC, forms a stable heterodimer with METTL14 localised at nuclear speckles to facilitate efficient m6A deposition.^21^, ^22^ METTL14, although lacking a catalytic domain and enzymatic activity, acts as a synergistic partner for METTL3, aiding in its interaction with RNA substrates through structural support.^21^, ^22^ WTAP is crucial for the nuclear speckle localisation of the METTL3/METTL14 heterodimer, thereby enhancing catalytic activity.^23^ Recent research has suggested that the cleaved form of METTL3 (residues 239−580) plays a vital role in recruiting WTAP efficiently into the MTC.^24^ METTL16 is responsible for catalysing the m6A methylation of U6 snRNA, lncRNAs and introns within pre‐mRNAs.^25^ Additionally, a protein known as VIRMA (or KIAA1429) facilitates site‐specific m6A deposition by bridging the METTL3/METTL14/WTAP complex with RNA substrates.^26^ RBM15 interacts with METTL3 and WTAP to determine the DRACH sites.^27^ Furthermore, ZC3H13 has also been demonstrated to enhance the nuclear localisation of WTAP.^28^ ZCCHC4, a specific m6A methyltransferase for 28S RNA, primarily mediates the m6A modification of this ribosomal RNA.^29^, ^30^ The E3 ubiquitin ligase HAKAI, a conserved component of the MTC, maintains the stability of other MTC components, ensuring proper m6A deposition.^31^

RNA m6A methylation is a reversible process governed by two proteins known as the FTO and ALKBH5, referred to as m6A ‘erasers’ due to their role in removing the methyl code from target mRNAs.^32^, ^33^ FTO, a member of the Fe (II) and oxoglutarate‐dependent AlkB oxygenase superfamily, was the first characterised m6A demethylase in 2011.^32^ Its discovery introduced the concept of reversible m6A modification.^32^ FTO is responsible for removing the internal methyl group, with a preference for demethylating m6Am, a less abundant in RNA modification.^32^ In 2013, ALKBH5 emerged as the second m6A demethylase. ALKBH5 directly converts m6A to adenosine and collaborates with FTO to maintain m6A levels.^33^

m6A modification significantly influences mRNA metabolism through interactions with m6A RNA‐binding proteins (RBPs), collectively known as readers. These reader proteins specifically recognise and bind to the m6A‐containing transcripts, thereby determining the fate of RNA^34^, ^35^, ^36^, ^37^ (Figure 1). The primary reader proteins include the YT521‐B homology (YTH) domain family proteins (YTHDF1/2/3), YTH domain‐containing proteins (YTHDC1/2), insulin‐like growth factor 2 mRNA‐binding proteins (IGF2BPs), heterogeneous nuclear ribonucleoproteins (hnRNPA2B1, hnRNPC and hnRNPG), eukaryotic initiation factor 3 (eIF3), FMRP and Prrc2a.^20^, ^35^, ^36^, ^37^ These reader proteins regulate various aspects of mRNA metabolism, encompassing translation, degradation, stability, nuclear export, microRNA processing and pre‐mRNA splicing^34^, ^35^, ^36^, ^37^ (Figure 1). For instance, YTHDF1, promotes translation initiation and elongation by interacting with translation initiation factor eIF3.^38^ It competes with another reader FMRP for binding to m6A‐modifed transcripts, thereby suppressing translation.^39^ Moreover, IGF2BPs and YTHDC2 enhance the translation of their target transcripts.^40^, ^41^ mRNA decay and stability are primarily regulated by reader proteins, with YTHDF2 being a notable participant, promoting mRNA decay in conjunction with the CCR4‐NOT deadenylase complex.^42^ YTHDF3, on the other hand, cooperates with YTHDF1 and YTHDF2 to regulate the translation or decay of m6A‐bearing RNA.^43^, ^44^ YTHDC2 and IGF2BPs are multifunctional reader proteins that influence mRNA decay and stability as well.^40^, ^41^ Additionally, the inhibition of YTHDC1 accelerates the degradation of specific nuclear mRNA.^45^ YTHDC1 also acts as a mediator of transcriptional repression through the lncRNA XIST,^46^ and facilitates the nuclear export of m6A‐modified mRNA in synergy with the export proteins SRSF3 and NXF1.^47^, ^48^ Prrc2a, a novel reader protein, stabilises targeted transcripts.^49^ Similar to other readers, FMRP also affects the decay and nuclear export of methylated mRNAs.^50^, ^51^ In addition to its impact on mature mRNAs, m6A readers modulate the biogenesis and function of ncRNAs. hnRNPA2B1 facilitates the processing of pri‐miRNAs to pre‐miRNAs by recruiting the DGCR8 protein.^52^ Recently, YTHDF3 has been reported to enhance circRNA biogenesis and control circ‐ZNF609 translation.^53^ YTHDC1, recognising m6A‐modified RNAs, induces transcriptional condensate formation and gene activation.^54^ Moreover, m6A readers participate in alternative splicing events, with YTHDC1 promoting exon inclusion in 293T cells by recruiting splicing factor SRSF3 or restricting SRSF10 recruitment in an m6A‐dependent manner.^18^ Similarly, in MIN6 cells, YTHDC1 regulates alternative splicing by interacting with SRSF3 and CPSF6 MIN6 cells.^55^ NKAP, identified as an m6A reader, can regulate the transcription termination site splicing event of m6A‐modified transcripts by recruiting the splicing factor SFPQ.^56^ NKAP also plays a role in miRNA maturation.^57^ The binding of hnRNPC and hnRNPG to targeted RNAs is regulated through a mechanism termed the ‘m6A switch’ which is involved in the splicing of m6A‐labelled mRNA.^58^, ^59^ Notably, hnRNPG cooperates with YTHDC1 to promote the exon inclusion of Itbp3bp and Nek1.^55^ In the following sections, we mainly summarise the potential relationship between m6A methylation and alternative splicing.

## 3 ALTERNATIVE SPLICING

The Human Genome Sequence Project has identified approximately 20 000 protein‐coding genes, a number significantly lower than the total count of human proteins.^60^ Alternative RNA splicing, a pivotal post‐transcriptional process primarily involving exon recombination, plays a critical role in generating multiple protein variants from a single multi‐exon gene.^61^, ^62^, ^63^, ^64^ This intricate process contributes significantly to the transcriptomic and proteomic diversity in mammals. In species characterised by high complexity, alternative splicing events occur with high frequency. It is estimated that over 95% of multi‐exon genes in humans undergo alternative splicing, which encompasses various processes such as exon shortening, elongation, exon inclusion or intron retention.^62^, ^63^ Precise regulation of alternative splicing necessitates a complex network of regulatory elements including splicing enhancers and silencers binding to specific pre‐mRNA sequences, either enhancing or inhibiting the splicing process.^62^, ^63^, ^64^ Furthermore, numerous RBPs and splicing factors act as key regulators, determining splice sites selection and influencing exon inclusion or exclusion.^62^, ^63^, ^64^ The meticulous regulation ensures the production of specific isoforms in a cell‐ or tissue‐specific manner, thereby enabling the generation of diverse protein variants with distinct functions.

### 3.1 Mechanisms of pre‐mRNA splicing

**FIGURE 2:**
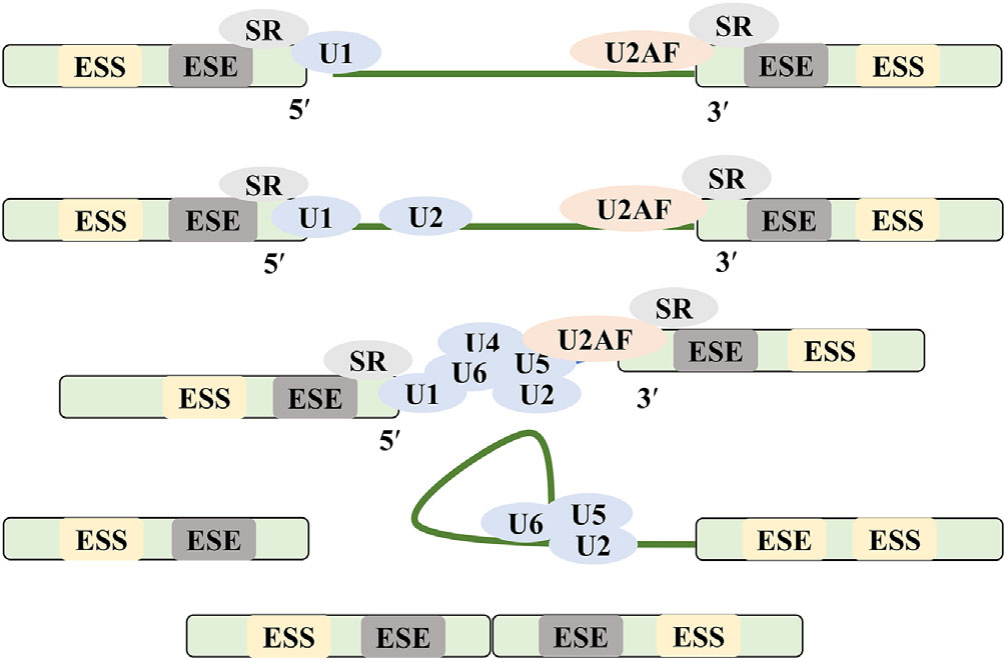
Schematic representation of pre‐mRNA splicing. The spliceosome assembly is a critical and complex process necessary for splicing reactions. The spliceosomal complexes are composed of small nuclear ribonucleoprotein particles (U1‐5 snRNPs), pre‐mRNA and various constitutive and ancillary proteins. Following two successive transesterification reactions that orchestrate intron removal and exon ligation, the mature mRNA is produced.

Canonical splicing regulation can be categorised into four phases: spliceosome assembly, catalytic activation, catalytic reaction and spliceosome disassembly.^11^, ^65^ Spliceosome assembly is a multi‐step and indispensable process for splicing reactions, characterised by a macromolecule complex comprising small nuclear ribonucleoprotein particles (snRNPs, U1, U2, U4/U6 and U5) and over 150 constitutive and ancillary proteins.^65^, ^66^ During spliceosome assembly, the U1 snRNP initially interacts with the targeted pre‐mRNA through the consensus motif of CAGGURAGU located at the 5′ splice site.^67^ Subsequently, the U2AF65 subunit binds to the polypyrimidine sequence, while the U2AF35 subunit engages with the AG dinucleotide at the 3′ splice site, forming the heterodimer known as the U2 snRNP auxiliary factor.^68^ The U2 snRNP is recruited to the branch point of the pre‐mRNA, followed by the addition of U5 and U4/U6 tri‐snRNP into U1 snRNP. Multiple DEAH box RNA helicases then rearrange snRNP interactions in terms of conformation and composition to form the active spliceosome.^69^ snRNPs, in conjunction with other splicing factors, associate with pre‐mRNAs to create the spliceosome, which subsequently undergoes two successive transesterification reactions, leading to intron removal and exon ligation.^65^ In the 1st transesterification reaction, a lariat structure forms at the 3′ end of the exon, while the 2nd transesterification step releases the lariat, eliminates the intron and ligates the exon, culminating in the generation the mature mRNA^65^ (Figure 2).

### 3.2 Mechanisms of pre‐mRNA alternative splicing

**FIGURE 3:**
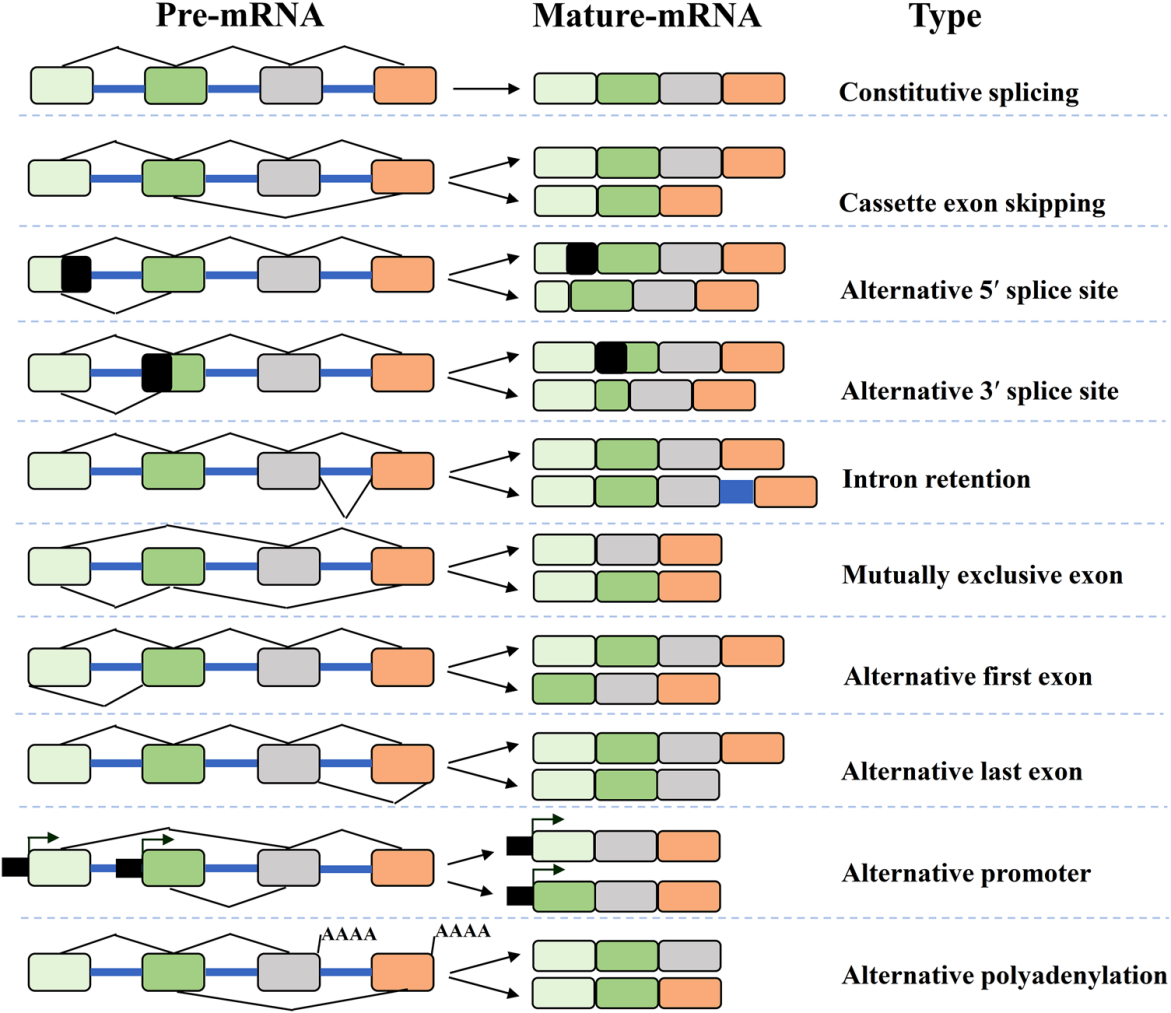
Schematic depiction of constitutive and alternative splicing patterns. Constitutive splicing mainly involves the removal of introns and the connection of exons to ensure the stability of gene expression. Alternative splicing events primarily involve different patterns: cassette exon skipping, mutually exclusive exon, alternative 5′ splice site, alternative 3′ splice site, intron retention, alternative first exon, alternative last exon, alternative promoter and alternative polyadenylation.

Alternative splicing encompasses various patterns, including (Cassette) exon skipping (SE), mutually exclusive exon (MXE), alternative donor site (A5’SS), alternative acceptor site (A3’SS), intron retention, alternative first exon, alternative last exon, alternative promoter and alternative polyadenylation (Figure 3). These patterns are regulated by trans‐acting factors that recognise cis‐acting splicing regulatory elements (SREs).^62^, ^64^, ^70^ SREs, including exonic splicing enhancer (ESE), intron splicing enhancer (ISE), as well as exonic splicing silencer, and intronic splicing silencer, can either promote or suppress RNA splicing depending on the interaction with trans‐acting factors.^60^, ^64^, ^70^ The trans‐acting factors that govern alternative splicing predominantly comprise splicing factors, which are RBPs. These splicing factors include including the arginine/serine‐rich (SR) proteins, heterogeneous ribonucleoproteins (hnRNP) proteins,^62^ and others. Among these factors, SR protein splicing factors (SRSF1‐12) have garnered significant attention recently owing to their crucial role in alternative splicing. Typically, SRSFs possess one or two RRM domains dictating their RNA‐binding specificity, in addition to an RS domain facilitating interaction with other SR proteins.^71^

SR proteins play a pivotal role in spliceosome assembly and rearrangement of spliceosomes, with their impact on splicing varying based on their binding site within the pre‐mRNA.^71^ Furthermore, SR proteins not only stimulate exon inclusion but also induce exon skipping through their interaction with the pre‐mRNA.^71^ Initially, SR proteins were recognised for binding to ESEs or exonic regions to enhance exon inclusion, as exemplified by SRSF1's role in regulating exon inclusion in the production of the Bcl‐xL isoform.^72^ However, subsequent research has unveiled that SR proteins can also induce exon skipping by altering their binding sites relative to the regulated splice site.^73^ Generally, SR proteins located within exons act as splicing enhancers, while those position in introns function as splicing inhibitors.^71^ Regarding spliceosome assembly, SRSF1, SRSF2 and SRSF6 participate in spliceosome formation by recruiting U1 snRNP to the 5′ splice site and U2 snRNP to the 3′ splice site.^74^

In contrast, hnRNP proteins, comprising 23 members, influence alternative splicing by changing their binding sites relative to the splice site being regulated.^75^ Unlike SR proteins, hnRNPs were initially identified as binding predominantly to bind to intronic regions. This interaction promotes exon skipping by disrupting the association between spliceosomes and splice sites, impeding exon recognition.^76^ For example, hnRNPA1's interaction with the flanking exon 9 of PKM leads to an increased skipping of exon 9 and the subsequent up‐regulation of the PKM2 isoform lacking exon 9.^77^ However, hnRNPs can also inhibit exon exclusion when binding to exonic regions.^78^ Furthermore, SR proteins and hnRNP proteins exhibit antagonistic activities, either promoting or inhibiting splicing.^79^ Consequently, modulation of the balance between SR and hnRNP proteins can impact alternative splicing in nuclear speckles.

Furthermore, numerous tissue‐specific splicing factors fall under the category of trans‐acting factors, such as RBM5, RBM24, Nova1 and QKI.^80^ These splicing factors contribute to the generation of tissue‐specific splicing events characterised by unique temporal and spatial profiles, thus enriching the diversity of cellular protein functions.^80^ For instance, RBM24 interacts with the ISE and promotes the retention of muscle‐specific exons in striated muscle.^81^ Notably, certain atypical splicing factors like U2AF65, SF3B3 and SF1 regulate alternative splicing by directly binding to pre‐mRNA rather than SREs.^82^, ^83^

## 4 THE REGULATION OF M6A MODIFICATION IN ALTERNATIVE SPLICING

As the most prevalent post‐transcriptional modification, m6A methylation exerts its influence across nearly every aspect of mRNA processing, including mRNA translation, decay, stability, alternative splicing and localisation.^21^ While previous research predominantly centred on m6A modification's role in regulating mRNA stability, degradation and translation, it has also been elucidated that m6A can regulate the alternative splicing of pre‐mRNA by recruiting splicing factors through m6A‐binding proteins^18^, ^84^ (Figure 1). In 2021, Wei G et al.^85^ demonstrated that approximately 10% of m6A signals are situated within alternative introns/exons in mouse embryonic stem cells, signifying potential crosstalk between the m6A machinery and RNA splicing. Moreover, early m6A deposition at 5′ and 3′ splice junctions enhances constitutive kinetics.^86^ Furthermore, m6A deposition within introns regulates alternative splicing events, underscoring the role of m6A modification in dictating the fate of nascent transcripts in terms of splicing kinetics and alternative splicing outcomes.^86^ More recently, Achour C et al.^87^ presented compelling evidence for m6A modification's role in controlling alternative splicing switches. They demonstrated that m6A signals are enriched at splice junctions of target transcripts and drive the expression of splicing factors through transcriptional regulator MYC, thereby contributing to the acquisition of malignant phenotypes in breast cancer cells.^87^ In concurrence, Jara‐Espejo M et al. first identified the co‐localisation of m6A and potential RNA G‐quadruplex structures at intron splice‐junctions.^88^ Furthermore, splicing factors have the ability to recognise consensus m6A motifs within introns proximal to 5′ and 3′ splice sites,^88^ suggesting that epitranscriptomic m6A modification may collaborate with RNA G‐quadruplex structures to regulate alternative splicing. These findings highlight the significant impact of m6A modification on the modulation of alternative splicing (Tables S1 and S2). In the subsequent sections, we provide an overview of the current understanding of how m6A influences alternative splicing in cancer and discuss the therapeutic potential of harnessing m6A‐dependent alternative splicing for cancer treatment.

### 4.1 The alternative splicing of m6A writer machinery

Recently, growing evidence has emerged linking m6A modification to alternative splicing. Current research on alternative splicing regulated by m6A writers predominantly revolves around the methyltransferase METTL3. METTL3, as the core enzyme responsible for m6A methylation, installs m6A on targeted RNAs, thereby influencing alternative splicing in various types of tumour cells.^34^, ^36^ A study by Dominissini D et al. reported that the down‐regulation of METTL3 in HepG2 cells results in the occurrence of differentially spliced exons and introns, with the deposition of m6A peaks through m6A‐seq.^89^ Further analysis using GO analysis revealed that silencing METTL3 induces cell apoptosis by modulating the p53 signalling through differentially spliced variants, such as MDM4, MDM2, FAS and BAX.^89^ These findings underscore the important role of m6A in alternative splicing events in hepatocellular carcinoma. Similarly, the depletion of METTL3 disrupts multiple pathways, including p53 signalling, VEGF signalling and cell cycle regulation, through aberrant alternative splicing in glioma stem‐like MGG8 cells.^90^ Subsequent studies have investigated the underlying mechanisms by which m6A modification promotes glioblastoma progression by regulating alternative splicing events.^91^ Genes with decreased expression are primarily involved in RNA processing and mRNA splicing in glioma stem cells lacking METTL3.^91^ The m6A modification increases the expression of SRSFs, particularly SRSF3, SRSF6 and SRSF11, in a YTHDC1‐dependent manner of nonsense‐mediated mRNA decay.^91^ This contributes to the production of anti‐apoptotic splice variants of BCL‐X and glioma stem cell‐promoting splice variants of NCOR2, ultimately facilitating to the growth and progression of glioblastoma.^91^ In bone mesenchymal stem cells, depletion of METTL3 not only inhibits VEGFA expression but also its splice variants, VEGFA‐164 and VEGFA‐188.^19^ FUBP1 has been identified as a ‘long tail’ cancer driver through a combinatorial CRISPR/Cas9 screen.^92^ FUBP1 cooperates with PTEN to reverse the malignant transformation of mammary epithelial cells.^92^ Further investigation revealed that blocking FUBP1 decreases the global m6A content by preventing the recruitment of METTL3, which is induced by PTEN.^92^ This leads to changes in the alternative splicing landscape and the expression of abnormal driver isoforms in breast cancer.^92^ In contrast, Achour C et al.^87^ proposed a contradictory viewpoint, wherein METTL3 promotes breast cancer progression by controlling tumour‐associated alternative splicing switch via the m6A deposition in splice site boundaries.^87^ Despite the conflicting evidence, these findings identify METTL3 as a crucial regulator of alternative splicing events in the breast cancer progression. The splicing switch of m6A modification has also been identified in oral squamous cell carcinoma.^93^ The m6A depositions on exon 3 and exon 5 maintain the structures of intron 2/exon 3, exon 3/intron 3 and exon 5/intron 5 of KRT4 pre‐mRNA by suppressing the binding of DGCR8 to the boundaries of intron 2/exon 3, exon 3/intron 3 and exon 5‐intron 5.^93^ These findings reveal that m6A methylation on the exon–intron boundary of pre‐mRNA hinders the intron splicing of pre‐mRNA by inhibiting the binding of DGCR8 to the exon–intron boundary.^93^ Furthermore, m6A modification not only directly influences the alternative splicing machinery, but also indirectly affects alternative splicing by regulating the abundance of various splicing factors in an m6A‐dependent manner in various cancers, including chronic lymphocytic leukaemia,^94^ leukaemia,^95^ breast invasive ductal carcinoma, colon adenocarcinoma, lung adenocarcinoma and gastric adenocarcinoma.^96^ These findings provide compelling evidence for the significant roles of m6A modification machinery In alternative splicing patterns.

Targeting METTL3 effectively generates differentially spliced variants in multiple primary cancers. Specifically, the silencing of METTL3 conferred by siRNAs or the treatment with the METTL3‐pharmacological inhibitor STM2457 has been shown to significantly alter the transcriptome and splicing patterns regulated by androgens in prostate cancer cells.^90^ The inhibition of METTL3 leads to the occurrence of numerous differential alternative splicing events, particularly exon skipping.^90^ This is accompanied by an increase in the ratio of androgen receptor‐FL to truncated androgen receptor variants, presenting a promising therapeutic target for manipulating androgen signalling in patients with prostate cancer.^90^ Importantly, METLL3, but not FTO, modulates the alternative splicing of the HPV16 E6/E7 mRNAs and gene expression in cervical cancer cells.^97^ This suggests a potential role of m6A modification in HPV‐induced cervical carcinogenesis.^97^ Additionally, the knockdown of METTL3 results in the alternative splicing of 1803 genes, with significant enrichment of cell cycle‐related functions in osteosarcoma cells.^98^ Further exploration reveals that 69.2−86.7% of these alternatively spliced genes exhibit m6A deposition near alternative splicing sites, along with the enrichment of 19 RBPs.^98^ These findings indicate that m6A‐dependent alternative splicing may be a critical factor in driving osteosarcoma progression. Collectively, these results demonstrate the relevance of alternative splicing events mediated by m6A modification in the initiation and progression of cancers.

The global view of MeRIP‐seq results of transcriptome‐wide m6A modification, demonstrates that METTL3‐dependent splicing is observed not only in tumour cells but also in non‐tumour cells (Tables S1 and S2). Furthermore, the inactivation of METTL3 and subsequent reversal of m6A modification lead to aberrant splicing events, predominantly characterised by exon skipping.^99^ These splicing events are enriched in the synapse‐associated pathway.^99^ Moreover, the depletion of METTL3 promotes exon 21 skipping of Grin1, resulting in a significant increase in intracellular calcium levels and subsequently inducing apoptosis in cerebellar granule cells.^99^ Furthermore, the loss of METTL3 has been shown to hinder the differentiation of spermatogonial cells and the onset of meiosis in germ cells, mainly by regulating the alternative splicing of genes involved in spermatogenesis through m6A modification.^100^ Additionally, lipopolysaccharides have been observed to stimulate the expression of METTL3 in dental pulp cells, providing a new perspective on the role of epitranscriptomic regulation in the inflammatory response.^101^ Further studies have revealed that the depletion of METTL3 modulates the alternative splicing of MyD88 and, as a result, leads to increased expression of the MyD88 S isoform, which inhibits the inflammatory response induced by lipopolysaccharide in dental pulp cells.^101^ Overall, these findings demonstrate that the m6A‐dependent alternative splicing patterns are critical for the effect of tumour pathophysiology and patient outcomes and the regulation of a wide spectrum of cellular and physiological processes.

Considering the diverse functions of m6A modification in mRNA metabolism, the dTAG degron system has been utilised to degrade METTL3 rapidly and efficiently, elucidating the discrimination of direct and indirect effects of METTL3.^85^ The findings reveal that the acute depletion of METTL3 specifically affects the inclusions of alternative introns and exons of nascent RNA in mouse embryonic stem cells.^85^ Moreover, the CRISPR‐mediated genome‐editing system is highly beneficial for characterising the m6A modification, highlighting the important mechanical role of m6A in alternative splicing events.^102^ Furthermore, the CRISPR/Cas9‐mediated cleavage of snoRNA SNORD75 in Gas5 led to the generation of exon 3–5 skipping in 293FT‐75‐2 cells, largely attributed to the disruption of the binding of the METTL3/METTL14 complex to SNORD75.^102^ A targeted RNA methylation system, composed of dCas13–M3nls and dCas13–M3M14nes, has been developed using the CRISPR–Cas13 tool combined with METTL3 and (or) METTL14.^103^ The RNA methylation system effectively manipulates the installation of m6A on diverse RNA targets, thereby modulating RNA abundance and alternative splicing.^103^ Additionally, the dCas13–M3nls system, when transfected into HEK293T cells with dCas13–M3nls,  results in alternative splicing events, such as the exclusion of Brd8 exon 21 and the inclusion of Znf638 exon 2.^103^ These findings shed light on our growing understanding of m6A in alternative splicing and provide a novel strategy for identifying the alternative splicing of m6A machinery. However, the use of targeted RNA m6A systems for the programmable manipulation of alternative splicing events in tumour treatment remains insufficiently explored.

Drosophila has emerged as a powerful in vivo model for dissecting the molecular mechanisms underlying m6A‐dependent alternative splicing.^104^, ^105^ In 2016, Lence T et al.^105^ demonstrated the significant role of m6A in sex determination through alternative splicing regulation. They observed that the down‐regulation of Ime4 (METTL3 orthologue in drosophila) or dMettl14 led to alternative splicing at the 5′ splice site and intron retention, ultimately determining the sex of the drosophila.^105^ The targeting of m6A modification is associated with the inclusion of male‐specific exon 3 and the reduction of the female‐specific isoform, while the female‐specific isoform depends on m6A‐dependent exon 3 skipping of Sxl.^105^ Notably, the effect of m6A on sex determination by regulating the alternative splicing of the *Sxl* gene has been independently corroborated by various other studies.^104^, ^106^ Five years later, additional advancements have been made by other researchers in understanding the splicing events of the *Sxl* gene.^107^ Further evidence has been put forth wherein in females, Sxl recruits m6A writer components, which subsequently introduce m6A modification in exon 3 and neighbouring introns of the Sxl transcript.^107^ Following this, YTHDC1 interacts with m6A‐modified regions to induce exon 3 skipping by interacting with splicing factors.^107^ These studies collectively support the functional importance of m6A‐dependent alternative splicing of Sxl in the sex determination of drosophila. However, it is yet to be determined if this mechanism is conserved in mammals.

Apart from METTL3, other methyltransferases also play a role in the regulation of alternative splicing. m6A modification is essential for the maturation of oligodendrocyte lineage cells and the myelination of the central nervous system by abating METTL14.^108^ METTL14 ablation inhibits the maturation of oligodendrocytes and impairs the thickness of the myelin sheath, accompanied by a decrease in myelinated axons.^108^ Mechanistically, METTL14 ablated mutants regulate oligodendrocyte transcripts and alternative splicing events of numerous RNA transcripts.^108^ Specifically, the ablation affects 1372 splicing events in 364 genes, such as Ptprz, in oligodendrocyte precursor cells.^108^ Additionally, in oligodendrocytes, it impacts 1930 splicing events in 485 genes, such as Nfasc.^108^ MAT2A has been characterised by encoding SAM synthetase. In a 2017 study, it was reported that METTL16, a conserved U6 snRNA methyltransferase, interacts with the hairpin regions of MAT2A 3′ UTR.^109^ This interaction is essential for inducing intron retention through the METTL16‐VCR domains, supporting the role of METTL3 as a central factor in maintaining SAM homeostasis.^109^ However, a more recent study in 2021 reported that METT‐10, the METTL16 orthologue in Caenorhabd *elegans*, regulates the homeostasis of SAM synthetase by regulating the alternative splicing of SAMS‐3 and SAMS‐4, which is accompanied by nonsense‐mediated mRNA decay induced by m6A modification.^110^ Recently, the mechanisms by which METT‐10 and human METTL16 induce m6A modification at the 3′ splice site of SAMS genes, which play a role in regulating SAM homeostasis, have been extensively explored.^111^  Studies reveal that although METT‐10 and METTL16 regulate SAM homeostasis through distinct regulatory mechanisms mediated by alternative splicing, the targeted RNAs that undergo m6A methylation are well‐conserved between *C. elegans* and *Homo sapiens*.^111^

WTAP has been implicated in regulating the alternative splicing of pre‐mRNA, such as Sxl, transformer and Ultrabithorax.^112^ The depletion of the drosophila homolog of WTAP, hFL(2)D, has been shown to affect the alternative 3′ splice sites of transformer pre‐mRNAs, which are substrates of Sxl regulation. This suggests that hFL(2)D has a regulatory role in sex determination.^112^ Additionally, FL(2)d promotes the skipping of exon 3 to maintain female identity and the inclusion of the small internal exons of Ultrabithorax.^113^ Furthermore, FL(2)d also interacts with early‐acting general splicing regulators, including U2AF38,  U170K, U2AF50 and Snf.^113^ Notably, Sxl has been co‐immunoprecipitated by Fl(2), with Fl(2)d regulating alternative splicing in an Sxl‐dependent manner.^113^ Therefore, Fl(2)d has been hypothesised to regulate splice‐site selection in the first splicing reaction with spliceosome assembly in an Sxl‐dependent manner. The role of WTAP in splicing events has been examined in mammalian cells.^114^ WTAP protein degradation induced by IGF‐1 through the nuclear 26S proteasome pathway leads to changes in the splicing switch of survival factor survivin in human vascular smooth muscle cells.^114^ Specifically, the antiapoptotic variant survivin increases and the proapoptotic survivin‐2B variant decreases.^114^ These findings highlight the clear linkage between the nuclear accumulation of WTAP and the balance of survivin isoforms. Although WTAP has been linked to the splicing switch, it is yet to be established whether the effect is dependent on methyltransferase activity mediated by WATP.

As a member of the split‐ends family of proteins, RBM15 plays a role in RNA metabolism including alternative splicing. RBM15 interacts with the intronic regions of specific pre‐mRNAs, namely GATA1, RUNX1, TAL1 and c‐MPL, to directly regulate their alternative splicing.^115^ This regulation is achieved by recruiting the intron‐binding splicing factor SF3B1. Through this mechanism, RBM15 contributes to the megakaryocyte differentiation and maturation of human primary cells.^115^ However, it remains unclear whether RBM15 controls the alternative splicing in an m6A‐dependent manner.

### 4.2 The alternative splicing of m6A eraser machinery

Evidence suggests that that m6A demethylases participate in the modulation of alternative splicing, particularly in response to various cellular conditions and infections. Virus infections, in particular, have highlighted the connection between m6A modification and alternative splicing. Using MeRIP‐seq, researchers have observed a decrease in m6A peaks in the last exon of CIRBP mRNA following Flaviviridae infection in Huh7 cells.^116^ Importantly, this infection‐induced reduction in m6A modification of CIRBP transcripts modulates their alternative splicing, resulting in the down‐regulation of the long CIRBP isoform with intron retention, while the short isoform remains unaffected.^116^ The present review presents compelling evidence for the impact of m6A modification on viral infection through alternative splicing. In the case of the demethylase ALKBH5, it has been observed to promote the production of HPV16 E6 mRNA with intron retention and contributes to the exon skipping of the late L1 mRNA in C33A2 cells.^97^ ALKBH5's removal of m6A modification is responsible for the correct splicing of longer transcripts in pachytene spermatocytes and round spermatids.^117^ However, the knockout of ALKBH5 in spermatogenic cells leads to an increased number of splicing events in longer transcripts, resulting in impaired male fertility and spermatogenesis.^117^

Interestingly, the ALKBH5‐dependent alternative splicing machinery has also been elucidated. Through the use of rMATS, a total of 90 differential alternative splicing events (A5SS, A3SS, MXE, RI and SE) are identified between PaCa‐2 cells overexpressing ALKBH5.^118^ The integration of RNA‐seq and MeRIP‐seq data allowed researchers to screen for hypo‐methylation and altered splicing events, ultimately identifying SLC25A37 as the only gene exhibiting significant changes in m6A peaks and splicing events. This also involved two hypo‐methylated m6A regions containing A5SS and A3SS events.^118^ Furthermore, they confirmed that ALKBH5 modulates the RNA splicing of SLC25A37 to generate isoform 1−4 through an m6A‐dependent mechanism, affecting iron metabolism in pancreatic ductal adenocarcinoma.^118^ Additionally, ALKBH5‐mediated m6A demethylation reduces the mRNA stability of CELF2, a splicing factor involved in mRNA splicing, translation and editing, thus highlighting the indirect effect of ALKBH5 on splicing events in pancreatic cancer.^119^ CELF2 controls the alternative splicing of CD44, resulting in the production of different CD44 isoforms through exon skipping.^119^ Consequently, CELF2 promotes the conversion of CD44s to CD44v isoform, which acts as a regulator that protects against the progression of pancreatic cancer through the endoplasmic reticulum‐associated degradation signalling pathway.^119^ However, knowledge of the mechanistic relationship between ALKBH5 and alternative splicing in human carcinogenesis remains limited.

FTO, another m6A demethylase, also plays a role in alternative splicing events. Blocking FTO activity has been demonstrated to significantly increase the inclusion of exon 6 in Runx1t1 pre‐mRNA, resulting in the production of the splice isoform Runx1t1‐L^exon6+^, while the Runx1t1‐S^exon6−^ isoform almost completely disappears in adipocytes.^120^ Studies also further explore the mechanisms by which the FTO‐mediated removal of m6A hinders the recruitment of SRSF2, wherein it leads to the exon 6 skipping in Runx1t1 and subsequently promoting the differentiation of pre‐adipocytes in mice.^120^ Similarly, Zhao X et al.^121^ also demonstrated that the depletion of FTO enhances the ability of SRSF2 protein to recognise exon splicing enhancers, thereby promoting the exon 6 skipping of Runx1t1 in mouse pre‐adipocytes. Collectively, these findings underscore the significance of the FTO‐dependent alternative splicing machinery in m6A demethylation‐dependent pathways, acting as a crucial regulatory mechanism during adipogenesis. In human 293T cells, FTO was observed to be more prone to interact with the intronic regions of pre‐mRNA, as well as with the alternative splicing exons and poly(A) sites located nearby.^122^ Furthermore, the deficiency of FTO leads to widespread changes in splicing events, with a particular increase in exon skipping.^122^ Interestingly, this exon skipping pattern is reversed after the knockdown of METTL3, another protein involved in RNA splicing, in 293T cells.^122^ Similar to m6A methyltransferases, the demethylase FTO has been demonstrated to regulate alternative splicing events through modulating and recruiting specific splicing factors to targeted mRNAs.

### 4.3 The role of m6A reader in alternative splicing

**FIGURE 4:**
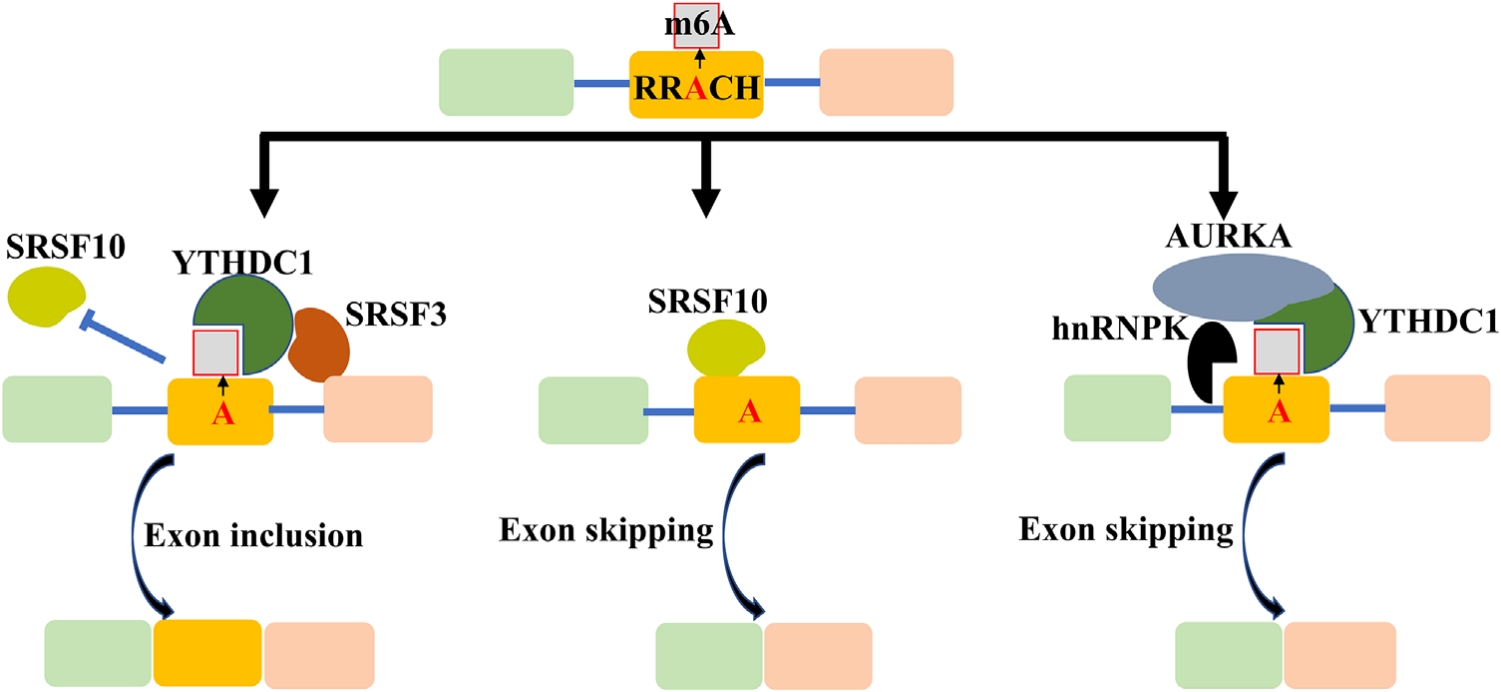
The mechanism of YTHDC1 in regulating alternative splicing. YTHDC1 selectively recruits SRSF3 over SRSF10 to facilitate the inclusion of specific exons in m6A‐modified mRNAs. However, this preference is disrupted when the m6A modification disappears. In addition, AURKA, which is transported into the nucleus recruits hnRNPK to YTHDC1, leading to exon skipping event.

Mounting evidence strongly supports the critical involvement of m6A readers in the regulation of alternative splicing, often acting in synergy with SRSFs. Among these readers, YTHDC1 emerges as the most extensively studied m6A reader in the context of splicing regulation.^18^, ^48^, ^84^, ^123^ It is now widely recognised that YTHDC1, in an m6A‐dependent manner, recruits specific splicing factors to m6A‐modified sites, thereby influencing alternative splicing events^18^, ^48^, ^84^ (Figure 4). YTHDC1, for instance, promotes exon inclusion in m6A‐modified mRNAs by recruiting SRSF3 while counteracting the mRNA binding of SRSF10 in 293T cells.^18^ An RNA pull‐down assay demonstrated that YTHDC1 can competitively bind with both SRSF10 and SRSF3. The m6A modification is indispensable for facilitating the nuclear speckle localisation, mRNA binding and alternative splicing by SRSF3 while inhibiting these functions when SRSF10 is involved^18^ (Figure 4). When m6A methylation is reversed or YTHDC1 is disrupted, SRSF10 promotes exon skipping in the targeted mRNA.^18^ Furthermore, recent studies indicate that YTHDC1 regulates the alternative splicing of lncHOXB‐AS3 through an m6A‐dependent mechanism.^123^ This regulation leads to an increase in the expression of lncHOXB‐AS3 spliceosome NR_033205.1, which, in turn, induces self‐renewal in self‐renewal of leukaemic stem cells and accelerates the progression of acute myeloid leukaemia.^123^ These studies provide compelling evidence on the regulatory role of m6A‐YTHDC1 in terms of alternative splicing, facilitating interactions between *trans*‐ and *cis*‐regulatory elements.

In human lung cancer cells, m6A‐YTHDC1 promotes exon 3 skipping, resulting in the production of the tumour‐promoting RBM4‐S^exon3−^ variant by recruiting hnRNPK mediated by AURKA. The RBM4‐S^exon3−^ variant is also known to play a tumour‐promoting role in lung cancer.^48^ YTHDC1 also plays a pivotal role in regulating the alternative splicing of HIV‐1 RNAs during HIV‐1 infection.^124^ YTHDC1's significance extends to oocyte development and embryonic development in mice, where its inactivation leads to significant alternative splicing events, such as the retention of introns in Phf1.^84^ Hu Y et al , investigated the underlying mechanisms by which YTHDC1 regulates mouse oocyte development.^125^ They observed that KIAA1429 knockout down‐regulated the m6A levels, primarily affecting the alternative splicing of mRNA involved in oogenesis.^125^ KIAA1429 promotes this alternative splicing through the recruitment of SRSF3 by YTHDC1, leading to exon inclusion.^125^ YTHDC1 also promotes the retention of E6‐encoding introns, inducing the production of full‐length E6 mRNA in human cells.^97^ However, the specific mechanism by which this occurs is yet to be fully elucidated.^97^ Additionally, m6A‐YTHDC1 is involved in back‐splicing events, a form of alternative splicing,^53^ promoting the biogenesis of circ‐ZNF609 through the back‐splicing reaction in an m6A‐dependent manner, albeit with no effect on its export or stability.^53^

IGF2BPs, another class of m6A readers, have been shown to affect tumour progression through alternative splicing.^126^, ^127^ Using eCLIP analysis, Tran TM et al.^127^ revealed that IGF2BP3 significantly influences the alternative splicing of numerous transcripts, with 261 splicing events in CD11b^+^ cells and 97 splicing events in Lin cells. The depletion of IGF2BP3 results in to decreased expression of both full‐length variants and alternatively spliced variants (shorter variant) for Cd69, Hoxa7 and Hoxa9, suggesting that IGF2BP3 serves as a critical regulator and a potential therapeutic target for MLL‐Af4‐driven leukaemia.^127^ In hepatocellular carcinoma, 53 IGF2BP3‐associated alternative splicing events have been identified,^126^ suggesting that IGF2BP3 may promote hepatocellular carcinoma progression through alternative splicing events that enrich multiple oncologic pathways.^126^ Similarly, IGF2BP3 affects 121 alternative splicing events involving cassette exon, 5pMXE and 3pMXE, with 61 specific alternative splicing genes in lung cancer.^128^ Specifically, IGF2BP3 regulates the alternative splicing patterns of PKM genes, known for aberrant splicing in various cancers, potentially promoting lung tumourigenesis and offering a novel therapeutic target for patients with lung cancer.^128^ Additionally, alternative splicing mediated by IGF2BP1 also plays a role in the progression of lung cancer.^129^ In coordination with other splicing factors, IGF2BP1 promotes the expression of the ncIMPAD1‐203 variant through the alternative splicing machinery, contributing to the epithelial‐mesenchymal transition process and EGFR‐TKI resistance in lung adenocarcinoma.^129^ These results collectively highlight the pivotal role of IGF2BPs in regulating alternative splicing patterns across various cancers.

**FIGURE 5:**
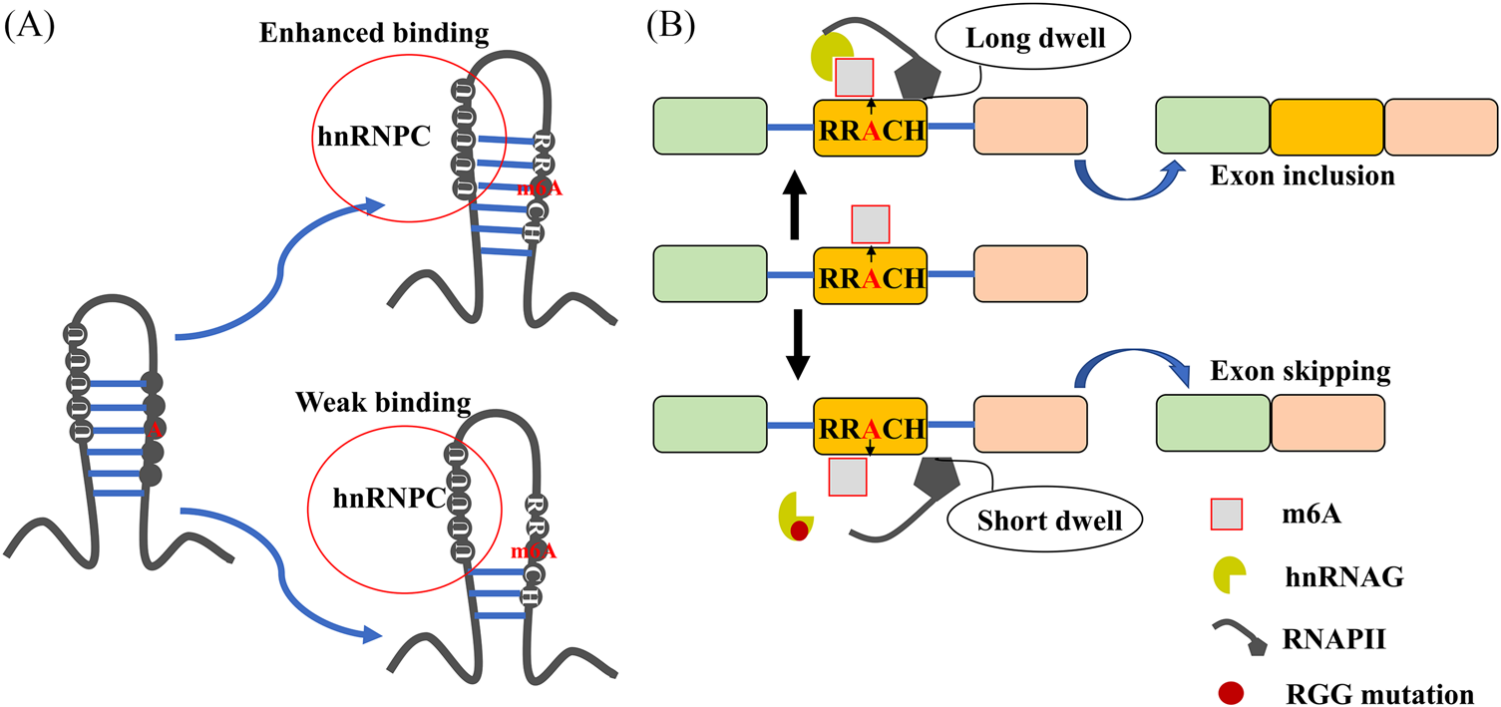
The mechanisms of hnRNPC and hnRNPG in regulating alternative splicing. (A) The m6A modification modulates the stability of the secondary structure of RNAs and facilitates the binding of the reader hnRNPC protein. m6A deposition at the hairpin‐stem of lncRNAs alters the structure stability and contributes to the interaction between hnRNPC and UUUUU motifs, which leads to s alternative splicing of the target RNAs. (B) The RRM and Arg‐Gly‐Gl (RGG) motifs of hnRNPG binds to m6A‐modified RNAs. In addition, it interacts with the phosphorylated C‐terminal domain of RNAP polymerase II (RNAPII) to facilitate its dwell at the exon–intron junction. This dwell increases the splice site utilisation, ultimately inducing exon inclusion. Conversely, mutations in the RGG motifs of hnRNPG induces an opposite effect.

The m6A reader hnRNPC binds to targeted mRNAs bearing m6A modification and regulates their alternative splicing through the m6A switch mechanism^130^, ^131^ (Figure 5). This switch modulates the stability of mRNA's secondary structure, thereby enhancing the interaction between protein and RNA^130^, ^131^ (Figure 5). For instance, m6A deposition at the hairpin‐stem of lncRNA MALAT1 alters the stability of its structure, increasing the accessibility of the U_5_‐tract bound by hnRNPC.^130^, ^131^ This ultimately regulates cellular processes including alternative splicing.^130^, ^131^ hnRNPC‐dependent alternative splicing accelerates the liver metastasis of pancreatic ductal adenocarcinoma.^132^ Specifically, hnRNPC facilitates the alternative splicing of TAF8 by binding to TAF8 mRNA, resulting in the production of the pro‐metastatic TAF8 S isoform in pancreatic ductal adenocarcinoma.^132^ Interestingly, mutation of the m6A sites within TAF8 disrupts the interaction between hnRNPC and the TAF8 transcript, leading to an increase in the anti‐metastatic TAF8 L isoform and a decrease in the TAF8 S isoform, thereby limiting the metastatic potential of cancer cells.^132^ The m6A switch mechanism serves as a foundation for understanding the alternative splicing regulated by m6A modification, offering insights into how splicing factors recognise and bind to m6A‐marked RNAs, thereby regulating alternative splicing.

Similar to hnRNPC, the m6A reader hnRNPG selectively binds to AGG[m6A]C motifs within the lncRNA MALAT1 hairpin structure through its C‐terminal low‐complexity region^133^ (Figure 5). The m6A methylation of MALAT1 enhances the accessibility of these motifs, facilitating hnRNPG interactions.^133^ hnRNPG primarily regulates alternative splicing by preferentially binding to m6A‐modified mRNAs, influencing the alternative splicing of nearby exons.^133^ Zhou KI, provided further insights into the mechanism of hnRNPG‐dependent alternative splicing.^59^ They discovered that hnRNPG interacts with m6A‐modified transcript and RNA polymerase II through its RRM and Arg‐Gly‐Gl motifs in the low‐complexity region. This interaction regulates the occupancy of RNA polymerase II and promotes the inclusion of exons^59^ (Figure 5). This study unveils a previously unidentified role of co‐transcriptional m6A in the regulation of splicing, mediated by hnRNPG^59^ (Figure 5).

hnRNPA2B1, another m6A reader and splicing factor, directly recognises the consensus m6A “RGAC” motif to regulate alternative splicing.^52^ In MDA‐MB‐231 cells, the depletion of hnRNPA2B1 has similar effects on alternative splicing as the depletion of METTL3,^52^ implying that hnRNPA2B1 serves as an executor for m6A‐dependent alternative splicing.^52^ hnRNPA2B1‐dependent alternative splicing events have been observed in multiple human cancers, involving both m6A‐dependent and m6A‐independent pathways.^134^ GO analyses have shown that hnRNPA2B1 significantly influences alternative splicing events in colon cancer cells that overexpress hnRNPA2B1, suggesting a crucial role in colon cancer progression.^134^ In gastric cancer cells, hnRNPA2B1 binds to the exon 4 of BIRC5 pre‐mRNA adjacent to 3′ UTR, regulating its alternative splicing.^135^ This binding results in an increase in the pro‐cancer BIRC5‐202 isoform and a decrease in the anti‐cancer BIRC5‐203 isoform, promoting gastric cancer progression and chemotherapy resistance.^135^ hnRNPA2B1 stabilised by Linc01232 enhances the expression of the full‐length A‐Raf isoform while reducing the short A‐Raf isoform through alternative splicing.^136^ This process promotes the metastasis of pancreatic cancer by activating the MAPK signalling pathway.^136^ Additionally, hnRNPA2B1 facilitates exon 11 skipping of MST1R pre‐mRNA, increasing the RONΔ165 isoform in head and neck cancers.^137^ This isoform activates the Akt/PKB signalling, ultimately leading to epithelial‐to‐mesenchymal transition in head and neck cancer.^137^ It is important to note that as an m6A reader protein, hnRNPA2B1 itself is a splicing factor responsible for regulating splicing events. However, further research is required to determine whether hnRNPA2B1‐dependent alternative splicing events are associated with m6A modification.

Recent research has unveiled NKAP as an m6A reader that directly interacts with the “RGm6AC” site on SLC7A11 mRNA.^56^ This interaction leads to the splicing event of its transcription termination site and the retention of the last exon by recruiting the splicing factor SFPQ, thereby protecting glioblastoma cells from ferroptosis.^56^ This discovery suggests the possibility of identifying more reader proteins with splicing functions in the future. Further investigation is also necessary to determine if other known m6A reading proteins also possess similar splicing functions.

## 5 ALTERNATIVE SPLICING REGULATES M6A MODIFICATION

Notably, alternative splicing events have the capacity to influence m6A modification and regulators (Table S3). The knockout of METTL3 does not fully reverse mRNA m6A methylation owing to the presence of spliced METTL3 variants in various cellular contexts, including mouse embryonic stem cells, U2OS cells and MEFs.^138^ These spliced variants of METTL3 effectively bypass CRISPR/Cas9‐induced mutations in METTL3, encoding catalytically active METTL3.^138^ Xu RY et al.^139^ also revealed the existence of spliced METTL3 variants in hepatocellular carcinoma. They found that these alternatively spliced variants of METTL3 can be detected in various human tissues.^139^ One such variant, termed METTL3‐D, undergoes alternative 3′ splicing events, resulting in a variant characterised by a shorter exon 4 and retention of introns 8 and 9, ultimately leading to decreased cellular m6A modification.^139^ In contrast, the canonical full‐length METTL3‐A isoform positively correlates with hepatocellular carcinoma progression, fostering increased cell proliferation, migration and invasion.^139^ Conversely, the METTL3‐D isoform appears to suppress cell proliferation and metastasis in hepatocellular carcinoma by decreasing the level of m6A modification.^139^ Furthermore, METTL3 isoforms with intron 8 and 9 retention have been identified in several human tissues.^140^ The splicing pattern that retains introns in METTL3 inhibits transcript export with intron retention in the cytoplasm, leading to a reduction in METTL3 expression.^140^ Similarly, Chen S et al.^141^ presented similar evidence for alternative splicing of METTL14 pre‐mRNA. They observed that CLK1‐mediated phosphorylation of SRSF5 at Serine 250 enhances the enrichment of SRSF5 in the pre‐mRNA of METTL14.^141^ This phosphorylation event subsequently inhibits exon 10 skipping in METTL14, resulting in an up‐regulation of the METTL14‐L^exon10+^ variant and a down‐regulation of the METTL14‐L^exon10−^ variant in pancreatic cancer.^141^ Importantly, the retention of exon 10 in METTL14 leads to an elevation in global m6A content and accelerates metastasis in pancreatic cancer.^141^ Furthermore, m6A peaks have been identified spanning exon 11 and intron 11 of the *YTHDC1* gene, situated in close proximity to the 5′ splice sites.^85^ Acute depletion of METTL3 has been demonstrated to increase YTHDC1 transcript levels and disrupt the alternative splicing of YTHDC1 in mouse embryonic stem cells.^85^ These findings collectively suggest that alternative splicing plays a significant role in the autoregulation of the m6A machinery. Consequently, this intricate interplay between m6A modification and alternative splicing not only highlights their mutual influence but also provides novel mechanistic insights into the regulatory mechanisms governing epitranscriptomic modulators.

## 6 CLINICAL IMPLICATIONS OF M6A METHYLATION IN ALTERNATIVE SPLICING REGULATION

The emerging roles of m6A modification in the regulation of alternative splicing are garnering increasing attention, particularly within the context of various diseases, especially cancers. Targeting the m6A machinery involved in alternative splicing offers promising avenues for both diagnostic and therapeutic interventions in cancer. Importantly, m6A‐dependent alternative splicing has been associated with cancer prognosis, presenting compelling evidence for prognostic and therapeutic stratification. In low‐grade glioma, for example, as many as 3272 alternative splicing events are regulated by the m6A regulator.^142^ Furthermore, m6A‐related alternative splicing plays a role in glioma progression through its influence on the immune‐microenvironment, making it a potential biomarker for prognostic assessment and personalised treatment strategies in patients with glioma.^142^, ^143^ Similarly, in oesophageal carcinoma, a negative correlation between m6A patterns and alternative splicing features in individual patients has been observed.^144^ Likewise, the involvement of m6A‐dependent alternative splicing and its regulatory players in clinical features suggests their potential as prognostic biomarkers for patients with non‐small cell lung cancer.^145^ These findings underscore the clinical relevance of alternative splicing in the context of m6A modification, and the alternative splicing of the m6A methylation machinery may represent an uncharted pathway in mediating tumour progression. Furthermore, m6A‐related alternative splicing events may prove to be potent prognostic biomarkers for patients with cancer, with the potential for constructing prognostic risk signatures based on m6A regulator‐induced alternative splicing patterns.

Moreover, m6A‐dependent alternative splicing has been implicated in influencing tumour initiation and progression. m6A modification also mediates chemotherapy resistance of tumour cells through alternative splicing events. For example, in pancreatic cancer cells, SRSF3 enhances resistance to gemcitabine chemotherapy by up‐regulating the expression of the lncRNA ANRIL‐L isoform through the inclusion of exon 1.^146^ Notably, m6A modification of ANRIL is essential for the SRSF3‐mediated inclusion of exon 1.^146^ Conversely, the alternative splicing of m6A modification machinery contributes to anti‐tumour effects. Baicalin hydrate, a natural compound with anti‐tumour activity, has been demonstrated to effectively inhibit the growth of nasopharyngeal carcinoma. This inhibitory effect is achieved by increasing the ratio of alternative splicing to constitutive splicing in Suv39H1 pre‐mRNA through the activation of m6A methylation.^147^ The underlying mechanism involves Baicalin hydrate‐induced up‐regulation of METTL3 and METTL4, which, in turn, promote m6A methylation at splice sites within the Suv39H1 transcript.^147^ Another notable example involves m6A modification influencing the glioma stem cell phenotype and apoptosis in glioblastoma cells. Here, m6A modification contributes to the increase in the NCOR2α isoform and a concomitant decrease in the NCOR2α isoform through the YTHDC1‐dependent alternative splicing machinery, thereby sustaining glioma stem cell stemness and reducing apoptosis in glioblastoma cells.^91^ These findings collectively underline the pivotal role of m6A‐dependent alternative splicing in regulating diverse tumourigenic phenotypes. Therefore, targeting m6A‐dependent alternative splicing holds significant promise as a therapeutic strategy in cancer treatment.

## 7 CONCLUSIONS AND PERSPECTIVES

As the most prevalent and abundant RNA modification in eukaryotes, m6A methylation has been revealed to exert significant effects on various stages of mRNA metabolism. The expanding body of evidence underscores the pivotal role of the m6A regulatory network in post‐transcriptional regulation, cellular and biological processes and human diseases.^1^, ^21^, ^148^ While earlier research primarily concentrated on the regulatory role of m6A modification in mRNA stability, degradation and translation, recent advancements have shed light on the interplay between m6A modification and RNA splicing.^17^, ^48^, ^88^, ^104^, ^117^, ^145^ Alternative splicing of pre‐mRNA is a critical post‐transcriptional process that governs the complex transcriptomic and proteomic landscape of mammals. Given the emerging significance of m6A modification in alternative splicing regulation, there exists a tremendous opportunity to enrich our comprehension of species complexity through the lens of m6A and to further deepen our mechanistic insights into m6A modification. This review aims to unravel the potential interaction between the m6A modification and alternative splicing, emphasising the strong connection between RNA epitranscriptomic modification and genomic intricacy.

m6A modification, serving as a double‐edged sword, wields both promoting and restraining influences on alternative splicing events, which play a critical role in various biological processes, such as sex determination, reproductive development, inflammatory response and adipogenesis.^48^, ^84^, ^99^, ^132^ The dysregulation of m6A‐dependent alternative splicing has been identified in numerous human diseases, with a particular emphasis on cancer. m6A modification can function as a splicing switch, controlling the fate of splicing events. m6A reader proteins regulate alternative splicing by recruiting a spectrum of splicing factors that recognise and bind to m6A consensus motifs proximal to splice junction.^16^ Meanwhile, m6A modification also exerts regulatory effects on splicing factors, such as SRSF3, SRSF6 and SRSF11.^91^ It is noteworthy that the m6A reader YTHDC1, an arbiter of splicing events, undergoes alternative splicing in response to m6A modification stimuli.^53^ This underscores the role of alternative splicing in the autoregulation of the m6A machinery.

m6A modification is associated with carcinogenesis and tumour progression. Targeting m6A modification offers promising strategies for combating tumours.^1^ Alternative splicing events are prevalent in nearly all human cancers and contribute to tumour progression by modulating various malignant phenotypes of tumour cells.^80^ The involvement of m6A modification in alternative splicing has been implicated in tumour initiation and progression, providing cancer cells with unique splicing profiles and features through alternative splicing regulation. The integration of alternative splicing and m6A modification sets the stage for novel prognostic models and therapeutic targets, offering personalised approaches to cancer treatment.

However, despite significant strides in understanding m6A modification and splicing regulation, much remains to be explored. Recent research by Luo Z et al.^149^ has revealed that exon–intron boundaries impede m6A modification within approximately 100 nucleotides of splice sites. Coincidentally, Uzonyi A et al.,^150^ in 2023, discovered that exon junction complexes actively exclude m6A deposition from splice site proximity. These complexes act as barriers to the enrichment of m6A marks near exon junctions.^151^ Consequently, the realm of alternative splicing in m6A modification machinery becomes increasingly intricate. Thus, identifying splice sites within m6A‐modified transcripts could pave the way for a deeper understanding of m6A‐dependent alternative splicing regulation. It remains unclear whether m6A readers operate independently or collaborate with each other in splicing regulation, as multiple splicing factors competitively bind to a given m6A reader to either promote or inhibit splicing events. The multifunctionality of m6A readers poses another challenge, necessitating further investigation. For instance, the reader hnRNPA2B1 serves as both a splicing factor for multiple mRNA alternative splicing and an executor for m6A‐dependent alternative splicing effects. However, it remains unclear whether hnRNPA2B1 regulates splicing events through m6A‐dependent or ‐independent mechanisms. While several inhibitors have been developed to target m6A regulators, their applicability to alternative splicing necessitates further exploration. Furthermore, apart from METTL3, the roles of other m6A regulators remain poorly understood. m6A‐involved alternative splicing continues to present itself as a complex and enigmatic landscape, of course, further study is urgently needed for the rosy scenario.

## CONFLICT OF INTEREST STATEMENT

The authors declare that they have no conflicts of interest.

## Supporting information

supporting information

supporting information

supporting information
