# Supplementary material for: Crosstalk between m6A modification and alternative splicing during cancer progression
Source: Clin Transl Med. 2023 Oct 18;13(10):e1460. doi: 10.1002/ctm2.1460 (PMC10583157; doi:10.1002/ctm2.1460)
Supplement: Supplementary file 1 — supporting information [file CTM2-13-e1460-s002.docx]

**Table 1. The function and mechanisms of m6A-mediated alternative splicing in tumor cells**

| Type | Regulator | Target gene | Mechanism | Biological function |
| --- | --- | --- | --- | --- |
|  | m6A regulator |  | m6A-related alternative splicing gene signature. | Mediates tumor progression through the immune-microenvironment and serves as a viable biological marker for patients with low-grade glioma^[^[^1^](#_ENREF_1)^]^. |
|  |  |  | m6A-related alternative splicing gene signature. | Predicts non-small cell lung cancer prognosis^[^[^2^](#_ENREF_2)^]^. |
|  |  |  | m6A clusters within individual tumors could predict alternative splicing. | May guide prognostic assessments of glioma prognosis and promote the use of effective strategies^[^[^3^](#_ENREF_3)^]^. |
|  |  |  | m6A score model is negatively correlated with alternative splicing risk model. | Affects the occurrence of these key alternative splicing events in esophageal carcinoma^[^[^4^](#_ENREF_4)^]^. |
| m6A writers | METTL3 | BCL | METTL3 leads to the production of anti-apoptotic splice variants of BCL-X. | Inhibits glioblastoma cell apoptosis^[^[^5^](#_ENREF_5)^]^. |
|  |  | NCOR2 | METTL3 reduces the expression of NCOR2α isoform. | Promotes glioblastoma cell proliferation^[^[^5^](#_ENREF_5)^]^. |
|  |  | Androgen receptor | Inhibition of METTL3 leads to the occurrence of numerous differential alternative splicing events, specifically exon skipping. | Presents a promising therapeutic target for prostate cancer patients^[^[^6^](#_ENREF_6)^]^. |
|  |  |  | METTL3 modulates p53 signaling via splicing. | Inhibits HepG2 cell apoptosis^[^[^7^](#_ENREF_7)^]^. |
|  |  | HPV16 E1 | METTL3 contributes to the inclusion of E1-intron on HPV16 mRNA. | Studes in tonsillar cancer cell line HN26^[^[^8^](#_ENREF_8)^]^. |
|  |  | HPV16 L1 | METTL3 promotes the exon inclusion of late L1. | Studies in tonsillar cancer cell line HN26^[^[^8^](#_ENREF_8)^]^. |
|  |  | lncRNA ANRIL | m6A modification of ANRIL is essential for the SRSF3-mediated inclusion of exon 1. | Promotes resistance of pancreatic cancer to gemcitabine^[^[^9^](#_ENREF_9)^]^. |
|  |  | MYC | m6A modification enriches in splice-junctions of MYC and induces MYC-driven the expression of splicing factors. | Contributes to the acquisition of the malignant phenotypes of breast cancer cells^[^[^10^](#_ENREF_10)^]^. |
|  |  |  | METTL3 leads to the changes in the alternative splicing landscape. | Produces the necessary panoply of contributors for breast cancer^[^[^11^](#_ENREF_11)^]^. |
|  |  | Cell cycle-related genes | METTL3 knockdown regulates the alternatively splicing of the cell cycle-related genes. | Plays a significant role in cancer development^[^[^12^](#_ENREF_12)^]^. |
|  |  | KRT4 | METTL3 and METTL14 suppresses the binding of DGCR8 to exon-intron boundaries in KRT4 pre-mRNA to prevente intron splicing of KRT4 pre-mRNA. | Provides potential therapeutic targets for oral squamous cell carcinoma^[^[^13^](#_ENREF_13)^]^. |
| m6A eraser | ALKBH5 | HPV E6 | ALKBH5 promotes the intron retention of HPV16 E6 pre-mRNAs. | Studies in tonsillar cancer cell line HN26^[^[^8^](#_ENREF_8)^]^. |
|  |  | HPV16 L1 | ALKBH5 promotes the exon skipping of HPV16 L1. | Studies in tonsillar cancer cell line HN26^[^[^8^](#_ENREF_8)^]^. |
|  |  | CD44 | ALKBH5 promotes CD44 alternative splicing and increases CD44v isoform by regulating splicing factor CELF2. | Promotes pancreatic cancer progression^[^[^14^](#_ENREF_14)^]^. |
| m6A readers | YTHDC1 | RBM4 | YTHDC1 recruits hnRNPK mediated by AURKA to alter alternative splicing of RBM4 and increases the production of RBM4-S isoform. | Promotes lung cancer progression^[^[^15^](#_ENREF_15)^]^. |
|  |  | HPV16 E6/E7 | YTHDC1 suppresses HPV16 E6/E7 mRNA splicing. | Studies in tonsillar cancer cell line HN26^[^[^8^](#_ENREF_8)^]^. |
|  |  | HOXB-AS3 | YTHDC1 increases the expression of HOXB-AS3 isoform NR_033205.1 via the m6A modification of HOXB-AS3 pre-RNA. | Accelerates the self-renewal of the self-renewal of leukemic stem cells and promotes the progression of acute myeloid leukemia^[^[^16^](#_ENREF_16)^]^. |
|  | IGF2BP1 | IMPAD1 | IGF2BP1 participates in the alternative splicing of IMPAD1, leading to the IMPAD1-203 variants that it lacks exon 1, part of exon 2 and exon 5, but it contains extra nucleotides in exon 3′ spliced from intron 3. | Induces EMT process leading to EGFR-TKI resistance in lung cancer cells^[^[^17^](#_ENREF_17)^]^. |
|  | IGF2BP3 |  | It has been identified 53 IGF2BP3-associated alternative splicing events. | Trigger aberrant alternative splicing events, which might contribute to hepatocellular carcinoma progression^[^[^18^](#_ENREF_18)^]^. |
|  |  | PKM and BTF3 | IGF2BP3 decreases the alternative splicing of PKM but increases the alternative splicing of BTF3. | May contributes to lung tumorigenesis by regulating the alternative splicing^[^[^19^](#_ENREF_19)^]^. |
|  |  | Hoxa9, Hoxa7, and Cd69 | GF2BP3 regulates the both alternatively spliced and full-length isoforms for Hoxa9, Hoxa7, and Cd69. | Highlights IGF2BP3 as a therapeutic target for MLL-AF4 leukemia^[^[^20^](#_ENREF_20)^]^. |
|  | hNRNPC | TAF8 | hNRNPC promotes the alternative splicing of TAF8 via binding to TAF8 mRNA and induces TAF8S isoform. | Promotes pancreatic ductal adenocarcinoma metastasis^[^[^21^](#_ENREF_21)^]^. |
|  |  | lncMALAT1 | hNRNPC binds to lncRNA MALAT1 through the U5-tract. | Regulates cellular processes including alternative splicing in HeLa cells^[^[^22^](#_ENREF_22)^]^. |
|  | hNRNPA2B1 |  | hnRNPA2B1 upregulation results in multiple gene alternative splicing. | Suggests the crucial role of hnRNPA2B1 in the progression of colon cancer^[^[^23^](#_ENREF_23)^]^. |
|  | NKAP | SLC7A11 | NKAP binds to the m6A-modified SLC7A11 to induce the retention of the last exon by the recruitment of splicing factor SFPQ. | Protects glioblastoma cells from ferroptosis^[^[^24^](#_ENREF_24)^]^. |

**References**

1. Maimaiti A, Tuersunniyazi A, Meng X, Pei Y, Ji W, Feng Z, Jiang L, Wang Z, Kasimu M, Wang Y, Shi X. N6-methyladenosine RNA methylation regulator-related alternative splicing gene signature as prognostic predictor and in immune microenvironment characterization of patients with low-grade glioma. Front Genet, 2022, 13(872186.

2. Zhao Z, Cai Q, Zhang P, He B, Peng X, Tu G, Peng W, Wang L, Yu F, Wang X. N6-Methyladenosine RNA Methylation Regulator-Related Alternative Splicing (AS) Gene Signature Predicts Non-Small Cell Lung Cancer Prognosis. Front Mol Biosci, 2021, 8(657087.

3. Zhao B, Xiang Z, Wu B, Zhang X, Feng N, Wei Y, Zhang W. Use of Novel m6A Regulator-mediated Methylation Modification Patterns in Distinct Tumor Microenvironment Profiles to Identify and Predict Glioma Prognosis and Progression, T-cell Dysfunction, and Clinical Response to ICI Immunotherapy. Curr Pharm Des, 2023, 29(1): 60-78.

4. Kong L, Gao F, Zhao F, Xia R, Cai C, Wang W, Huang D, Li Z, Yi Q, Zang C, Pu Y. Profiling the m(6)A-regulated RNA expression patterns and alternative splicing features in esophageal carcinoma. Genes Dis, 2023, 10(5): 1812-5.

5. Li F, Yi Y, Miao Y, Long W, Long T, Chen S, Cheng W, Zou C, Zheng Y, Wu X, Ding J, Zhu K, Chen D, Xu Q, Wang J, Liu Q, Zhi F, Ren J, Cao Q, Zhao W. N(6)-Methyladenosine Modulates Nonsense-Mediated mRNA Decay in Human Glioblastoma. Cancer Res, 2019, 79(22): 5785-98.

6. Visvanathan A, Patil V, Abdulla S, Hoheisel JD, Somasundaram K. N(6)-Methyladenosine Landscape of Glioma Stem-Like Cells: METTL3 Is Essential for the Expression of Actively Transcribed Genes and Sustenance of the Oncogenic Signaling. Genes (Basel), 2019, 10(2).

7. Dominissini D, Moshitch-Moshkovitz S, Schwartz S, Salmon-Divon M, Ungar L, Osenberg S, Cesarkas K, Jacob-Hirsch J, Amariglio N, Kupiec M, Sorek R, Rechavi G. Topology of the human and mouse m6A RNA methylomes revealed by m6A-seq. Nature, 2012, 485(7397): 201-6.

8. Cui X, Nilsson K, Kajitani N, Schwartz S. Overexpression of m6A-factors METTL3, ALKBH5, and YTHDC1 alters HPV16 mRNA splicing. Virus Genes, 2022, 58(2): 98-112.

9. Wang ZW, Pan JJ, Hu JF, Zhang JQ, Huang L, Huang Y, Liao CY, Yang C, Chen ZW, Wang YD, Shen BY, Tian YF, Chen S. SRSF3-mediated regulation of N6-methyladenosine modification-related lncRNA ANRIL splicing promotes resistance of pancreatic cancer to gemcitabine. Cell Rep, 2022, 39(6): 110813.

10. Achour C, Bhattarai DP, Groza P, Roman AC, Aguilo F. METTL3 regulates breast cancer-associated alternative splicing switches. Oncogene, 2023, 42(12): 911-25.

11. Elman JS, Ni TK, Mengwasser KE, Jin D, Wronski A, Elledge SJ, Kuperwasser C. Identification of FUBP1 as a Long Tail Cancer Driver and Widespread Regulator of Tumor Suppressor and Oncogene Alternative Splicing. Cell Rep, 2019, 28(13): 3435-49 e5.

12. Kim Y, Shin S, Kwon S, Moon K, Baek SV, Jo A, Kim HS, Hwang GH, Bae S, Kim YH, Cho SY, Oh JM. METTL3 regulates alternative splicing of cell cycle-related genes via crosstalk between mRNA m(6)A modifications and splicing factors. Am J Cancer Res, 2023, 13(4): 1443-56.

13. Li X, Fang J, Tao X, Xia J, Cheng B, Wang Y. Splice site m(6)A methylation prevents binding of DGCR8 to suppress KRT4 pre-mRNA splicing in oral squamous cell carcinoma. PeerJ, 2023, 11(e14824.

14. Lai S, Wang Y, Li T, Dong Y, Lin Y, Wang L, Weng S, Zhang X, Lin C. N6-methyladenosine-mediated CELF2 regulates CD44 alternative splicing affecting tumorigenesis via ERAD pathway in pancreatic cancer. Cell Biosci, 2022, 12(1): 125.

15. Li S, Qi Y, Yu J, Hao Y, He B, Zhang M, Dai Z, Jiang T, Li S, Huang F, Chen N, Wang J, Yang M, Liang D, An F, Zhao J, Fan W, Pan Y, Deng Z, Luo Y, Guo T, Peng F, Hou Z, Wang C, Zheng F, Xu L, Xu J, Wen Q, Jin B, Wang Y, Liu Q. Nuclear Aurora kinase A switches m(6)A reader YTHDC1 to enhance an oncogenic RNA splicing of tumor suppressor RBM4. Signal Transduct Target Ther, 2022, 7(1): 97.

16. Wu C, Cui J, Huo Y, Shi L, Wang C. Alternative splicing of HOXB-AS3 underlie the promoting effect of nuclear m6A reader YTHDC1 on the self-renewal of leukemic stem cells in acute myeloid leukemia. Int J Biol Macromol, 2023, 237(123990.

17. Chen QW, Cai QQ, Yang Y, Dong S, Liu YY, Chen ZY, Kang CL, Qi B, Dong YW, Wu W, Zhuang LP, Shen YH, Meng ZQ, Wu XZ. LncRNA BC promotes lung adenocarcinoma progression by modulating IMPAD1 alternative splicing. Clin Transl Med, 2023, 13(1): e1129.

18. Wang Y, Yang F, Shang J, He H, Yang Q. Integrative analysis reveals the prognostic value and functions of splicing factors implicated in hepatocellular carcinoma. Sci Rep, 2021, 11(1): 15175.

19. Xueqing H, Jun Z, Yueqiang J, Xin L, Liya H, Yuanyuan F, Yuting Z, Hao Z, Hua W, Jian L, Tiejun Y. IGF2BP3 May Contributes to Lung Tumorigenesis by Regulating the Alternative Splicing of PKM. Front Bioeng Biotechnol, 2020, 8(679.

20. Tran TM, Philipp J, Bassi JS, Nibber N, Draper JM, Lin TL, Palanichamy JK, Jaiswal AK, Silva O, Paing M, King J, Katzman S, Sanford JR, Rao DS. The RNA-binding protein IGF2BP3 is critical for MLL-AF4-mediated leukemogenesis. Leukemia, 2022, 36(1): 68-79.

21. Huang XT, Li JH, Zhu XX, Huang CS, Gao ZX, Xu QC, Zhao W, Yin XY. HNRNPC impedes m(6)A-dependent anti-metastatic alternative splicing events in pancreatic ductal adenocarcinoma. Cancer Lett, 2021, 518(196-206.

22. Zhou KI, Parisien M, Dai Q, Liu N, Diatchenko L, Sachleben JR, Pan T. N(6)-Methyladenosine Modification in a Long Noncoding RNA Hairpin Predisposes Its Conformation to Protein Binding. J Mol Biol, 2016, 428(5 Pt A): 822-33.

23. Tang J, Chen Z, Wang Q, Hao W, Gao WQ, Xu H. hnRNPA2B1 Promotes Colon Cancer Progression via the MAPK Pathway. Front Genet, 2021, 12(666451.

24. Sun S, Gao T, Pang B, Su X, Guo C, Zhang R, Pang Q. RNA binding protein NKAP protects glioblastoma cells from ferroptosis by promoting SLC7A11 mRNA splicing in an m(6)A-dependent manner. Cell Death Dis, 2022, 13(1): 73.
