# Supplementary material for: Crosstalk between m6A modification and alternative splicing during cancer progression
Source: Clin Transl Med. 2023 Oct 18;13(10):e1460. doi: 10.1002/ctm2.1460 (PMC10583157; doi:10.1002/ctm2.1460)
Supplement: Supplementary file 2 — supporting information [file CTM2-13-e1460-s001.docx]

**Table 2. The function and mechanisms of m6A-mediated alternative splicing in non-tumor cells**

| Type | Regulator | Target gene | Mechanism | Biological Behavior Changes |
| --- | --- | --- | --- | --- |
| m6A writers | METTL13 | Vegfa | METTL3 inhibits the expression of Vegfa and its splice variants (Vegfa-164 and Vegfa-188 but not Vegfa-120). | Affects osteogenic differentiation of bone marrow mesenchymal stem cells^[^[^1^](#_ENREF_1)^]^. |
|  |  | Grin1 | METTL3 suppress skipping exon21 of Grin1. | Inhibits cerebellar granule cells apoptosis. |
|  |  | Dazl, Sohlh1, Nasp and Cdk11b | METTL3 regulates the alternative splicing of genes functioning in spermatogenesis. | Regulates spermatogonial differentiation and initiation of meiosis^[^[^2^](#_ENREF_2)^]^. |
|  |  | MyD88 | METTL3 promotes the alternative splicing of MyD88 and upregulates MyD88S isoform. | Promotes lipopolysaccharide-induced inflammatory response of human dental pulp cells^[^[^3^](#_ENREF_3)^]^. |
|  |  | ZNF280D, PHE4DIP, and NEB | METTL3 regulates the alternative splicing of ZNF280D, PHE4DIP, and NEB. | Induces the conversion of oxidative fiber to glycolytic fiber^[^[^4^](#_ENREF_4)^]^. |
|  |  | Gas5 | METTL3-mediated m6A methylation affect lncRNA Gas5 skipping exon 3 to 5. | CRISPR Cas9 indirectly demonstrates the effect of METTL3 on alternative splicing^[^[^5^](#_ENREF_5)^]^. |
|  |  | Brd8 and Znf638 | CRISPR-Cas13 tool coupled with METTL3 and (or) METTL14 is capable of resulting the exclusion of Brd8 exon 21 and the inclusion of Znf638 exon 2. | Indicates that CRISPR-Cas13 tool system effectively manipulates the installation of m6A modifications^[^[^6^](#_ENREF_6)^]^. |
|  |  | Dazl | METTL3 promotes a longer transcript of Dazl. | Regulates spermatogonial differentiation and meiosis initiation^[^[^2^](#_ENREF_2)^]^. |
|  | Ime4 | Sxl | Ime4 promotes the skipping exon3 (male-specific exon) of Sxl pre-mRNA via YT521-B. | Determinates Drosophila sex^[^[^7^](#_ENREF_7)^]^. |
|  | METTL14 | Nfasc | METTL14 alters multiple gene alternative splicing including Nfasc. | Regulates the nodes of ranvier formation, oligodendrocyte maturation and central nervous system myelination^[^[^8^](#_ENREF_8)^]^. |
|  | METTL16 | MAT2A | Occupancy of METTL16 on hp1 promotes the retained intron of MAT2A. | Maintains the SAM homeostasis in human HEK293 cells^[^[^9^](#_ENREF_9)^]^. |
|  | METT-10 | SAM | METT-10 regulates the SAM homeostasis via modulating SAM pre-mRNA alternative splicing accompanied by nonsense-mediated decay of the pre-mRNAs. | Maintains the SAM homeostasis in in C. elegans^[^[^10^](#_ENREF_10)^]^. |
|  | WTAP | survivin | WTAP increases survivin-2B isoform via survivin pre-mRNA alternative splicing. | Suppresses human smooth muscle cell survival^[^[^11^](#_ENREF_11)^]^. |
|  |  |  | WTAP functions as a regulatory subunit of m6A methyltransferase complex. | Regulates gene expression and alternative splicing^[^[^12^](#_ENREF_12)^]^. |
|  | hFL(2)D | Sxl | fl(2)d promotes the exon 3 skipping of Sxl. | Regulates Drosophila sex determination^[^[^13^](#_ENREF_13)^]^. |
|  |  |  | fl(2)d affects alternative splicing of transformer pre-mRNA using alternative 3’ splice sites. |  |
|  |  |  | fl(2)d alters splice-site selection at an early step in spliceosome assembly in Sxl. |  |
|  | KIAA1429 |  | KIAA1429 promotes the target transcripts alternative splicing referring to exon inclusion through the recruitment of SRSF3 by YTHDC1. | Regulates mouse oogenesis^[^[^14^](#_ENREF_14)^]^. |
|  | METTL3 and METTL14 | CIRBP | Loss of m6A modification of CIRBP reduces the expression of its long isoform through changes in its interactions with splicing factors. | Affects flaviviridae infection^[^[^15^](#_ENREF_15)^]^. |
|  | FIONA1 | U6 snRNA | FIONA1 is required for Arabidopsis U6 snRNA m6A modification. | Determines the selection of degenerate 5' splice sites crucial to alternative splicing^[^[^16^](#_ENREF_16)^]^. |
| m6A eraser | ALKBH5 |  | ALKBH5 in the nuclei of spermatocytes and round spermatids is essential for correct splicing and the production of longer 3'-UTR mRNAs. | Regulates mouse spermiogenesis^[^[^17^](#_ENREF_17)^]^. |
|  | FTO | RUNX1T1 | FTO promotes RUNX1T1 skipping exon 6 via enhancing the ability of RNA binding to SRSF2. | Promotes adipogenesis and fat deposition in mice^[^[^18^](#_ENREF_18)^]^. |
|  |  |  | FTO depletion triggers exon skipping of pre-mRNA and this process depend on METTL3-mediated m6A modification. | Mediates the demethylation activity that influences several mRNA processing events^[^[^19^](#_ENREF_19)^]^. |
| m6A readers | YTHDC1 |  | YTHDC1 recruits SRSF3 to promote the exon inclusion or recruits SRSF10 to accelerate the exon skipping. | Indicates that YTHDC1 regulates mRNA splicing through recruiting and modulating pre-mRNA splicing factors^[^[^20^](#_ENREF_20)^]^. |
|  |  | HIV-1 | YTHDC1 may recruit splicing factors to regulate the alternative splicing of HIV-1 RNAs. | Demonstrates that m6A residues interact with cellular readers to positively affect HIV-1 mRNA function^[^[^21^](#_ENREF_21)^]^. |
|  |  |  | YTHDC1 deficiency results in multiple alternative splicing defects. | Regulates mouse oocyte development^[^[^22^](#_ENREF_22)^]^. |
|  |  |  | YTHDC1 recruits SRSF3 to promote the exon inclusion. | Regulates mouse oogenesis^[^[^14^](#_ENREF_14)^]^. |
|  |  | Pi4k2a | YTHDC1 promotes the intron retain of between Exons 9 and 10 of Pi4k2a. | Functions as a potential therapeutic target for PIP3-related diseases^[^[^23^](#_ENREF_23)^]^. |
|  |  | circ-ZNF609 | YTHDC1 regulates the back-splicing reaction in an m6A-dependent manner | Promotes the biogenesis of circ-ZNF609^[^[^24^](#_ENREF_24)^]^. |
|  |  | Itbp3bp and Nek1 | YTHDC1 depletion promotes the exon inclusion of Itbp3bp and Nek1 synergistically with hnRNPG. | Controls the regenerative ability of skeletal muscle stem cells by regulating mRNA splicing and nuclear export^[^[^25^](#_ENREF_25)^]^. |
|  | IGF2BP1 | ISCU | IGF2BP1 has a higher affinity for the mutant sequence to affect the splicing of the ISCU minigene. | Acts a potential factor in the pathology of hereditary myopathy with lactic acidosis^[^[^26^](#_ENREF_26)^]^. |
|  | IGF2BP2 | NFIC | IGF2BP2 promotes the skipping exons 9 and 10 in the NFIC gene and generates the protein CTF5. | Causes excessive granulosa cell proliferation in polycystic ovary syndrome^[^[^27^](#_ENREF_27)^]^. |
|  | hNRNPG |  | hNRNPG uses RGG motifs to interact with RNAPII. And it binds to m6A-modified nascent pre-mRNA, which affect RNAPII occupancy on exons and regulate exon inclusion. | Promotes the alternative splicing by m6A through the low-complexity protein hnRNPG^[^[^28^](#_ENREF_28)^]^. |
|  | hNRNPA2B1 |  | hNRNPA2B1 binds to m6A modification sites in pri-miRNA and modulates its alternative splicing which similar to METT13-mediated pri-miRNA alternative splicing. | Promotes pri-miRNA maturation^[^[^29^](#_ENREF_29)^]^. |
|  | hNRNPC |  | hNRNPC binds to target mRNAs to regulate its alternative splicing and gene expression. | Demonstrates the effects of m6A-switches on gene expression and RNA maturation^[^[^30^](#_ENREF_30)^]^. |

(Ime4: the METTL3 orthologue in Drosophila; METT-10: the METTL16 orthologue in C. elegans; hFL(2)D: the WTAP orthologue in Drosophila)

**References**

1. Tian C, Huang Y, Li Q, Feng Z, Xu Q. Mettl3 Regulates Osteogenic Differentiation and Alternative Splicing of Vegfa in Bone Marrow Mesenchymal Stem Cells. Int J Mol Sci, 2019, 20(3).

2. Xu K, Yang Y, Feng GH, Sun BF, Chen JQ, Li YF, Chen YS, Zhang XX, Wang CX, Jiang LY, Liu C, Zhang ZY, Wang XJ, Zhou Q, Yang YG, Li W. Mettl3-mediated m(6)A regulates spermatogonial differentiation and meiosis initiation. Cell Res, 2017, 27(9): 1100-14.

3. Feng Z, Li Q, Meng R, Yi B, Xu Q. METTL3 regulates alternative splicing of MyD88 upon the lipopolysaccharide-induced inflammatory response in human dental pulp cells. J Cell Mol Med, 2018, 22(5): 2558-68.

4. Tan B, Zeng J, Meng F, Wang S, Xiao L, Zhao X, Hong L, Zheng E, Wu Z, Li Z, Gu T. Comprehensive analysis of pre-mRNA alternative splicing regulated by m6A methylation in pig oxidative and glycolytic skeletal muscles. BMC Genomics, 2022, 23(1): 804.

5. Filippova JA, Matveeva AM, Zhuravlev ES, Balakhonova EA, Prokhorova DV, Malanin SJ, Shah Mahmud R, Grigoryeva TV, Anufrieva KS, Semenov DV, Vlassov VV, Stepanov GA. Are Small Nucleolar RNAs "CRISPRable"? A Report on Box C/D Small Nucleolar RNA Editing in Human Cells. Front Pharmacol, 2019, 10(1246.

6. Wilson C, Chen PJ, Miao Z, Liu DR. Programmable m(6)A modification of cellular RNAs with a Cas13-directed methyltransferase. Nat Biotechnol, 2020, 38(12): 1431-40.

7. Haussmann IU, Bodi Z, Sanchez-Moran E, Mongan NP, Archer N, Fray RG, Soller M. m(6)A potentiates Sxl alternative pre-mRNA splicing for robust Drosophila sex determination. Nature, 2016, 540(7632): 301-4.

8. Xu H, Dzhashiashvili Y, Shah A, Kunjamma RB, Weng YL, Elbaz B, Fei Q, Jones JS, Li YI, Zhuang X, Ming GL, He C, Popko B. m(6)A mRNA Methylation Is Essential for Oligodendrocyte Maturation and CNS Myelination. Neuron, 2020, 105(2): 293-309 e5.

9. Pendleton KE, Chen B, Liu K, Hunter OV, Xie Y, Tu BP, Conrad NK. The U6 snRNA m(6)A Methyltransferase METTL16 Regulates SAM Synthetase Intron Retention. Cell, 2017, 169(5): 824-35 e14.

10. Watabe E, Togo-Ohno M, Ishigami Y, Wani S, Hirota K, Kimura-Asami M, Hasan S, Takei S, Fukamizu A, Suzuki Y, Suzuki T, Kuroyanagi H. m(6) A-mediated alternative splicing coupled with nonsense-mediated mRNA decay regulates SAM synthetase homeostasis. EMBO J, 2021, 40(14): e106434.

11. Small TW, Pickering JG. Nuclear degradation of Wilms tumor 1-associating protein and survivin splice variant switching underlie IGF-1-mediated survival. J Biol Chem, 2009, 284(37): 24684-95.

12. Ping XL, Sun BF, Wang L, Xiao W, Yang X, Wang WJ, Adhikari S, Shi Y, Lv Y, Chen YS, Zhao X, Li A, Yang Y, Dahal U, Lou XM, Liu X, Huang J, Yuan WP, Zhu XF, Cheng T, Zhao YL, Wang X, Rendtlew Danielsen JM, Liu F, Yang YG. Mammalian WTAP is a regulatory subunit of the RNA N6-methyladenosine methyltransferase. Cell Res, 2014, 24(2): 177-89.

13. Ortega A, Niksic M, Bachi A, Wilm M, Sanchez L, Hastie N, Valcarcel J. Biochemical function of female-lethal (2)D/Wilms' tumor suppressor-1-associated proteins in alternative pre-mRNA splicing. J Biol Chem, 2003, 278(5): 3040-7.

14. Hu Y, Ouyang Z, Sui X, Qi M, Li M, He Y, Cao Y, Cao Q, Lu Q, Zhou S, Liu L, Liu L, Shen B, Shu W, Huo R. Oocyte competence is maintained by m(6)A methyltransferase KIAA1429-mediated RNA metabolism during mouse follicular development. Cell Death Differ, 2020, 27(8): 2468-83.

15. Gokhale NS, McIntyre ABR, Mattocks MD, Holley CL, Lazear HM, Mason CE, Horner SM. Altered m(6)A Modification of Specific Cellular Transcripts Affects Flaviviridae Infection. Mol Cell, 2020, 77(3): 542-55 e8.

16. Parker MT, Soanes BK, Kusakina J, Larrieu A, Knop K, Joy N, Breidenbach F, Sherwood AV, Barton GJ, Fica SM, Davies BH, Simpson GG. m(6)A modification of U6 snRNA modulates usage of two major classes of pre-mRNA 5' splice site. Elife, 2022, 11(

17. Tang C, Klukovich R, Peng H, Wang Z, Yu T, Zhang Y, Zheng H, Klungland A, Yan W. ALKBH5-dependent m6A demethylation controls splicing and stability of long 3'-UTR mRNAs in male germ cells. Proc Natl Acad Sci U S A, 2018, 115(2): E325-E33.

18. Azzam SK, Alsafar H, Sajini AA. FTO m6A Demethylase in Obesity and Cancer: Implications and Underlying Molecular Mechanisms. Int J Mol Sci, 2022, 23(7).

19. Bartosovic M, Molares HC, Gregorova P, Hrossova D, Kudla G, Vanacova S. N6-methyladenosine demethylase FTO targets pre-mRNAs and regulates alternative splicing and 3'-end processing. Nucleic Acids Res, 2017, 45(19): 11356-70.

20. Xiao W, Adhikari S, Dahal U, Chen YS, Hao YJ, Sun BF, Sun HY, Li A, Ping XL, Lai WY, Wang X, Ma HL, Huang CM, Yang Y, Huang N, Jiang GB, Wang HL, Zhou Q, Wang XJ, Zhao YL, Yang YG. Nuclear m(6)A Reader YTHDC1 Regulates mRNA Splicing. Mol Cell, 2016, 61(4): 507-19.

21. Tsai K, Bogerd HP, Kennedy EM, Emery A, Swanstrom R, Cullen BR. Epitranscriptomic addition of m(6)A regulates HIV-1 RNA stability and alternative splicing. Genes Dev, 2021, 35(13-14): 992-1004.

22. Kasowitz SD, Ma J, Anderson SJ, Leu NA, Xu Y, Gregory BD, Schultz RM, Wang PJ. Nuclear m6A reader YTHDC1 regulates alternative polyadenylation and splicing during mouse oocyte development. PLoS Genet, 2018, 14(5): e1007412.

23. Liu J, Zuo H, Wang Z, Wang W, Qian X, Xie Y, Peng D, Xie Y, Hong L, You W, Lou H, Luo G, Ren J, Shen B, Zheng J, Wang H, Ju Z. The m6A reader YTHDC1 regulates muscle stem cell proliferation via PI4K-Akt-mTOR signalling. Cell Prolif, 2023, 56(8): e13410.

24. Di Timoteo G, Dattilo D, Centron-Broco A, Colantoni A, Guarnacci M, Rossi F, Incarnato D, Oliviero S, Fatica A, Morlando M, Bozzoni I. Modulation of circRNA Metabolism by m(6)A Modification. Cell Rep, 2020, 31(6): 107641.

25. Qiao Y, Sun Q, Chen X, He L, Wang D, Su R, Xue Y, Sun H, Wang H. Nuclear m6A reader YTHDC1 promotes muscle stem cell activation/proliferation by regulating mRNA splicing and nuclear export. Elife, 2023, 12(

26. Nordin A, Larsson E, Holmberg M. The defective splicing caused by the ISCU intron mutation in patients with myopathy with lactic acidosis is repressed by PTBP1 but can be derepressed by IGF2BP1. Hum Mutat, 2012, 33(3): 467-70.

27. Zhao F, Wu L, Wang Q, Zhao X, Chen T, Yin C, Yan L, Yang X. Insulin-like growth factor 2 mRNA-binding protein 2-regulated alternative splicing of nuclear factor 1 C-type causes excessive granulosa cell proliferation in polycystic ovary syndrome. Cell Prolif, 2022, 55(4): e13216.

28. Zhou KI, Shi H, Lyu R, Wylder AC, Matuszek Z, Pan JN, He C, Parisien M, Pan T. Regulation of Co-transcriptional Pre-mRNA Splicing by m(6)A through the Low-Complexity Protein hnRNPG. Mol Cell, 2019, 76(1): 70-81 e9.

29. Alarcon CR, Goodarzi H, Lee H, Liu X, Tavazoie S, Tavazoie SF. HNRNPA2B1 Is a Mediator of m(6)A-Dependent Nuclear RNA Processing Events. Cell, 2015, 162(6): 1299-308.

30. Liu N, Dai Q, Zheng G, He C, Parisien M, Pan T. N(6)-methyladenosine-dependent RNA structural switches regulate RNA-protein interactions. Nature, 2015, 518(7540): 560-4.
