# Supplementary material for: Crosstalk between m6A modification and alternative splicing during cancer progression
Source: Clin Transl Med. 2023 Oct 18;13(10):e1460. doi: 10.1002/ctm2.1460 (PMC10583157; doi:10.1002/ctm2.1460)
Supplement: Supplementary file 3 — supporting information [file CTM2-13-e1460-s003.docx]

**Table 3. The function and mechanisms of alternative splicing regulating m6A regulators**

| m6A regulators | Alternative splicing | m6A modification | Biological function |
| --- | --- | --- | --- |
| METTL3 | Exon 2 and/or exon 4 skipping | Regulates m6A modification. | Spliced METTL3 can still lead to m6A modification^[^[^1^](#_ENREF_1)^]^. |
| METTL3 | Intron 8 and 9 inclusion | Decreases m6A modification. | Intron retention decreases METTL3 expression by inhibiting mRNA export to the cytoplasm^[^[^2^](#_ENREF_2)^]^ |
| METTL3 | 13 splice variants | Regulates m6A modification. | Variant METTL3 with a shorter exon 4 and retention of intron 8 and 9 suppresses cell proliferation and metastasis in hepatocellular carcinoma by decreasing the level of m6A modification^[^[^3^](#_ENREF_3)^]^. |
| METTL14 | Exon 10 inclusion | Increases m6A modification. | Promotes pancreatic cancer cells proliferation and metastasis^[^[^4^](#_ENREF_4)^]^. |
| WTAP | Intron 6 retention | Regulates m6A modification. | METTL3 enhances the inclusion of the intron-derived alternative last exon^[^[^5^](#_ENREF_5)^]^. |
| YTHDC1 | Intron 11 exclusion | - | Intronic m6A peaks located close to 5’ splice sites in YTHDC1 leads to alternative 5’ splice sites. Reversing m6A modification at intron 11 results in the alternatively spliced short isoform and more efficient YTHDC1 transcript^[^[^5^](#_ENREF_5)^]^. |

**References**

1. Poh HX, Mirza AH, Pickering BF, Jaffrey SR. Alternative splicing of METTL3 explains apparently METTL3-independent m6A modifications in mRNA. PLoS Biol, 2022, 20(7): e3001683.

2. Lee S, Jung H, Choi S, Cho N, Kim EM, Kim KK. Intron retention decreases METTL3 expression by inhibiting mRNA export to the cytoplasm. BMB Rep, 2023.

3. Xu RY, Ding Z, Zhao Q, Ke TY, Chen S, Wang XY, Wang YY, Sheng MF, Wang W, Long N, Shen YX, Xu YZ, Shao W. An Alternatively Spliced Variant of METTL3 Mediates Tumor Suppression in Hepatocellular Carcinoma. Genes (Basel), 2022, 13(4).

4. Chen S, Yang C, Wang ZW, Hu JF, Pan JJ, Liao CY, Zhang JQ, Chen JZ, Huang Y, Huang L, Zhan Q, Tian YF, Shen BY, Wang YD. CLK1/SRSF5 pathway induces aberrant exon skipping of METTL14 and Cyclin L2 and promotes growth and metastasis of pancreatic cancer. J Hematol Oncol, 2021, 14(1): 60.

5. Wei G, Almeida M, Pintacuda G, Coker H, Bowness JS, Ule J, Brockdorff N. Acute depletion of METTL3 implicates N (6)-methyladenosine in alternative intron/exon inclusion in the nascent transcriptome. Genome Res, 2021, 31(8): 1395-408.
